# Improving Cardiometabolic Health in Adolescent Girls With Elevated Anxiety: Web-Based Multisite, Pilot, and Feasibility Randomized Controlled Trial

**DOI:** 10.2196/82781

**Published:** 2026-09-17

**Authors:** Caitlin Edwards, Lisa Ranzenhofer, Jason Lavender, Lauren D Gulley, Stephen Aichele, Isabel Thorstad, Natalia Sanchez, Ruby Schrag, Zoe Sinkford, Jill Emerick, Victoria Thomas, Thomas Arnold, Jami Young, Denise Wilfley, Mark Haigney, Lauren Shomaker, Marian Tanofsky-Kraff

**Affiliations:** 1 Department of Human Development and Family Studies, College of Health and Human Services, Colorado State University, 410 W. Pitkin St, 1570 Campus Delivery, Fort Collins, CO, 80523, United States, 1 970-491-3217; 2 Endocrinology, Department of Pediatrics, University of Colorado Anschutz Medical Campus and Children’s Hospital Colorado, Aurora, CO, United States; 3 Department of Psychiatry, University at Buffalo Jacobs School of Medicine and Biomedical Sciences, Buffalo, NY, United States; 4 Department of Medicine, Military and Cardiovascular Outcomes Research (MiCOR), Uniformed Services University of the Health Sciences, Bethesda, MD, United States; 5 The Metis Foundation, San Antonio, TX, United States; 6 University of Colorado Anschutz Medical Campus, 6Barbara Davis Center for Diabetes, Aurora, CO, United States; 7 Department of Pediatrics, Uniformed Services University, Bethesda, MD, United States; 8 Children's Hospital of Philadelphia, University of Pennsylvania Perelman School of Medicine, Philadelphia, PA, United States; 9 Department of Psychiatry, Washington University in St. Louis, St. Louis, MO, United States; 10 Department of Medical and Clinical Psychology, Uniformed Services University, Bethesda, MD, United States

**Keywords:** anxiety, adolescents, cardiometabolic health, pilot trial, feasibility, interpersonal psychotherapy, cognitive behavioral therapy, eating behaviors

## Abstract

**Background:**

Anxiety is common in adolescents with elevated BMI (kg/m^2^), affecting 33%‐43% of adolescents with overweight or obesity (age- and sex-specific BMI ≥85th percentile ). Anxiety has been associated with poorer cardiometabolic health, and disinhibited eating in response to anxiety may be a key explanatory mechanism. Therefore, reducing anxiety in adolescents may improve disinhibited eating patterns and prevent worsening cardiometabolic health.

**Objective:**

This pilot study primarily aimed to assess the feasibility and acceptability of 2 virtual group psychological interventions, interpersonal psychotherapy (IPT) and cognitive behavioral therapy (CBT), in adolescent girls with elevated anxiety and above-average weight. Secondary aims were to describe changes in anxiety, eating behavior, and cardiometabolic health indices.

**Methods:**

This 2-site, 2-year, pilot and feasibility randomized controlled trial enrolled 12‐ to 17-year-old girls with elevated anxiety symptoms (State-Trait Anxiety Inventory for Children total score ≥32) and above-average weight (BMI ≥75th percentile for age and sex), who were in good general health and not concurrently receiving therapy or medication treatment. Girls were randomly assigned to 1 of 2 web-based arms: 12-week, psychologist-led, group IPT or CBT. In both arms, groups of 5‐7 adolescents met for 90 minutes on secure Zoom (Zoom Communications, Inc) once per week. Anxiety, disinhibited eating, BMI indices, blood pressure, lipids, hemoglobin A_1c_ (HbA_1c_), and fasting glucose were measured at baseline, 12-week follow-up, and 1-year follow-up. Feasibility of recruitment, retention, and study protocol, and acceptability and fidelity of IPT and CBT were evaluated.

**Results:**

Of 47 eligible adolescents, 40 (85%) enrolled. Enrollment of the total sample (n=40) was completed in 7 months. Retention was 85% (n=17) in IPT and 100% (n=20) in CBT at 12-week follow-up. Protocol adherence exceeded 96% for all intervals. Missing data varied, with higher rates for phlebotomy (up to 30%). Acceptability ratings were above-average for likability (mean 3.03, SD 1.14) and credibility (mean 3.26, SD 0.75; 0‐4 scale with 4=Highest) across arms. Most adolescents received ≥80% intervention dosage (IPT: 90%, CBT: 95%). Expert fidelity ratings of IPT were 78% (SD 11%) and CBT were 94% (SD 9%). From baseline to 1-year, IPT demonstrated improvements in general anxiety (95% CI −8.95 to −1.40), social anxiety (95% CI −12.37 to −1.88), and HbA_1c_ (95% CI −0.48 to −0.23); CBT demonstrated improvement in emotional eating (95% CI −1.18 to −0.18), and both arms demonstrated improvements in BMI percentile (effect sizes=−0.82 and −0.63, IPT and CBT, respectively).

**Conclusions:**

This pilot and feasibility trial provided initial support for the feasibility of recruitment and retention and acceptability of web-based group IPT and CBT, as well as yielded areas for optimizing protocol feasibility and intervention fidelity. These preliminary results inform the planning of larger-scale efficacy trials with powered samples to assess effects on BMI and cardiometabolic health indicators among adolescents with elevated anxiety and above-average weight.

## Introduction

### Overweight or Obesity in Adolescence

Elevated BMI (kg/m²) in adolescents is a serious public health concern. In the United States, about one-third of adolescents have overweight or obesity [1], and globally, adolescent obesity has increased by 1.5% annually over the past decade [2]. Overweight or obesity contributes to earlier manifestation and rising prevalence of cardiometabolic diseases such as type 2 diabetes and cardiovascular diseases [3,4]. Adolescents with overweight or obesity are more likely to develop insulin resistance, hypertension, dyslipidemia, and prediabetes [3,4]. Such cardiometabolic health concerns disproportionately impact individuals from historically disadvantaged identities [4,5], and rates are generally higher among girls than among boys [5]. Moreover, Hispanic, Latinx, and Black or African American individuals also experience higher rates of cardiometabolic and cardiovascular health problems across the lifespan, often beginning in adolescence [5,6], and resulting in a significant disease burden [2,6].

Adolescence is a sensitive period for the course of excess weight gain and cardiometabolic health [7], as there are significant changes in nutrition, sleep, and physical activity throughout adolescence [8]. Adolescence is also marked by physical changes in body composition that accompany puberty as well as psychosocial changes, including increases in perceived stress [9] and negative affect (eg, anxiety and depression) [10]. Rates of anxiety and depression are increasing among adolescents, with up to 60% of adolescents reporting symptoms of depression and 30% reporting anxiety [11]. Importantly, adolescents with elevated BMI are at higher risk for anxiety disorders, with 33%-43% of adolescents with overweight or obesity reporting elevated symptoms of anxiety [12-14]. Anxiety symptoms, especially those associated with social anxiety, commonly manifest during adolescence and are more prevalent in girls [15]. The dynamic physical and psychosocial transitions that characterize adolescence present both a period of heightened risk and an opportunity for impactful intervention to prevent excess weight gain and worsening cardiometabolic health by addressing anxiety.

### Cardiometabolic Disease Prevention

The typical behavioral approach to cardiometabolic disease prevention is characterized by one-size-fits-all intensive lifestyle treatment focused on decreasing caloric intake and increasing physical activity [16]. However, such approaches have faced challenges with attrition and sustained effectiveness in adolescents, necessitating more targeted and preventative approaches [16]. As an alternative, addressing psychosocial factors underlying eating and other health behaviors important for excess weight gain and cardiometabolic health may be particularly suitable for adolescents with above-average BMI.

Anxiety is posited to affect cardiometabolic health through anxiety-induced behaviors. Anxiety is associated with disinhibited eating (ie, the propensity to overeat in response to stimuli such as appetizing foods or negative emotions) [17-19]. Indeed, emotional dysregulation, such as anxiety, impacts eating decisions via reward circuitry and interoception; palatable foods become more salient during moments of negative affect [20]. Higher anxiety symptoms are also associated with more frequent disinhibited eating [21,22], especially loss-of-control eating [23,24], a disordered eating behavior characterized by a perceived inability to control intake regardless of the amount consumed [24,25]. Furthermore, disinhibited eating patterns, including loss of control and eating in response to negative affect, are associated with greater weight gain, adiposity, and higher insulin resistance in youth [23-26]. Thus, anxiety during adolescence can trigger disinhibited eating leading to excess weight, adiposity, and cardiometabolic health problems.

Frequent anxiety also activates stress-response biobehavioral systems, including the hypothalamic-pituitary-adrenal axis [27], sympathetic-adrenal-medullary system [28], and increased inflammatory response [29]. Repeated and sustained experiences of anxiety contribute to persistent hypothalamic-pituitary-adrenal activation, thereby increasing cortisol [30], which leads to visceral fat accumulation, increased gluconeogenesis, endothelial dysfunction, and dyslipidemia [30]. The ongoing activation of the sympathetic-adrenal-medullary system is associated with increased rates of hypertension, resting heart rate, and decreased heart rate variability [31,32]. Furthermore, chronic anxiety results in upregulated proinflammatory cytokines [33], thereby lowering insulin resistance, increasing atherogenesis, and impairing glucose control [33].

Treatment for anxiety has been shown to normalize stress physiology [34], improve health behaviors [35], and decrease allostatic load [30]. Moreover, psychological interventions to address both mental *and* physical health problems demonstrate greater acceptability than interventions that address mental *or* physical health concerns [36]. Therefore, anxiety represents a distinct treatment target for preventing further excess weight gain and cardiometabolic problems among adolescents with elevated anxiety and above-average BMI, particularly by improving disinhibited eating.

### Interventions

Cognitive behavioral therapy (CBT), the standard of care for adolescent anxiety [37,38], has also been shown to reduce disinhibited eating patterns in adolescents [39]. CBT is based on the premise that anxiety is sustained or exacerbated by the interplay of negative thoughts and behavioral avoidance. From this perspective, maladaptive responses to negative affect (eg, disinhibited eating) are learned behaviors that can be replaced with adaptive cognitive and behavioral coping strategies. CBT involves learning to identify and restructure negative thoughts and attitudes as well as using behavioral strategies focused on exposure to anxiety-inducing stimuli and rewards for nonavoidant coping [37,38]. Although CBT is a standard treatment for adolescent anxiety [37,38], its application to improving weight and cardiometabolic health outcomes in adolescents with elevated anxiety and above-average weight has not yet been tested.

Interpersonal psychotherapy (IPT) offers an alternative to CBT. IPT is grounded in interpersonal theory, which holds that social relationships drive negative affect and stress-related behavior [40]. IPT targets 4 interpersonal problem areas, including grief, role transitions, role disputes, and interpersonal difficulties, that sustain or worsen negative affect [40]. Through facilitated and targeted communication exercises, role-plays, and psychoeducation, IPT aims to improve relationships by reducing conflict, enhancing communication, and increasing meaningful relational support, thereby reducing negative affect and stress-related behaviors [40,41]. IPT has been shown to reduce excess weight and adiposity gain over 3 years compared with didactic controls among adolescent girls with elevated anxiety and above-average BMI [42]. Studies in adolescents with disinhibited eating suggest IPT’s promise for reducing loss-of-control eating [42], although IPT has yet to be tested with adolescents with anxiety symptoms specifically.

Extant research has compared the efficacy of IPT and CBT when treating adult depression in both randomized clinical trials [43] and in community settings [44], as well as when delivered via telehealth [45]. Generally, IPT and CBT have been found to be equally effective at treating both adult and adolescent anxiety and depression [43,44]. Indeed, IPT and CBT both are considered evidence-based treatments for adolescent mood disorders [45]. Moreover, IPT has been studied in adolescents with disinhibited eating with the aim to prevent excess weight [42,46] and CBT demonstrates clinically significant improvement for adolescents with eating disorders [47].

However, the *interpersonal* focus of IPT and the *intrapersonal* emphasis of CBT have not been specifically compared in adolescents with anxiety and above-average BMI. Both therapies could be expected to decrease anxiety symptoms and, in turn, reduce excess weight gain and associated cardiometabolic health indices by ameliorating disinhibited eating. Yet, to date, no research has been conducted comparing the efficacy of IPT versus CBT for weight and cardiometabolic health, nor are there data elucidating the potential differing mechanisms of change underlying IPT versus CBT.

However, before efficacy or mechanisms can be evaluated, feasibility and acceptability must be established in adolescents with elevated anxiety and above-average BMI. Multisite studies are ideal for maximizing external validity, timely recruitment of a targeted sample, and capacity for long-term follow-up [48]. Mental health interventions delivered via telehealth have been found to be effective in managing diverse mental health conditions among adolescents [49-52]. Additionally, virtually delivered group psychotherapy is a cost-effective means for increasing access to high-quality mental health care for harder-to-reach communities [53,54]. Virtual interventions have been found to reduce mental health stigma [52,53] and increase access to mental health treatment in historically underserved populations [52-54].

Therefore, we carried out a 2-site, pilot and feasibility randomized controlled trial of group IPT versus group CBT, delivered virtually, for adolescent girls with elevated anxiety and above-average BMI (institutional review board [IRB] number USUHS.2020‐048). In line with pilot study recommendations [55], main outcomes were the feasibility of recruitment, feasibility of research procedures, feasibility of retention, intervention fidelity, and acceptability of interventions.

## Methods

### Procedures

Enrollment for this pilot and feasibility randomized controlled trial was carried out at two sites: (1) the Uniformed Services University (USU) in Bethesda, Maryland, and (2) Colorado State University (CSU) in Fort Collins, Colorado. Participants were girls aged 12-17 years with elevated anxiety symptoms, as indicated by a total score of ≥32 on the State-Trait Anxiety Inventory for Children (STAI-C; [56]) and above-average BMI (≥75th percentile for age or sex). Exclusion criteria included any major medical conditions, pregnancy or breastfeeding, regular medication use likely to impact mood or weight (eg, insulin sensitizers, glucagon-like peptide-1 receptor agonists, antidepressants, and stimulants), current involvement in psychotherapy, and/or a psychiatric disorder or symptoms (eg, active suicidal ideation, behavior, or self-harm) that, in the opinion of the investigators, would impede competence, compliance, or otherwise hinder completion of the study. Exclusion criteria were chosen to ensure that adolescents who needed a higher level of care—beyond what could be provided in the context of a research study—were not included and any medications or interventions that could impact anxiety, eating behaviors, and/or cardiometabolic health were removed to mitigate confounding variables. If participants developed exclusionary criteria after randomization, they were withdrawn if participation was counter-indicated for safety or validity in the opinion of the investigators.

Recruitment took place from February 2021 to August 2021. Letters and flyers were sent to parents of girls aged 12-17 years who resided within 60 miles of USU or CSU; letters were also mailed to local pediatricians and family physicians. The study was advertised on school and parent email listserves, in local newspapers, and on social media. The target sample size was 40 participants (20 per site), in line with recommendations for pilot studies in which the primary effect size in future efficacy trials is expected to be small to moderate [57].

Adolescents and their parents initially completed a phone prescreening to estimate eligibility. Adolescents who were interested and appeared eligible were scheduled for an in-person screening and baseline data collection visit at either USU or CSU. The screening visit included written consent and assent, body measurements and fasting blood draw, brief psychiatric and medical health history with the participant and parent or guardian, clinical assessment interview, and surveys. Medical health history included age of menarche and menstrual cycle status. Parents completed questionnaires about parental education (highest education attained) and subjective socioeconomic status using the MacArthur Scale of Subjective Social Status [58].

Questionnaires, such as the family history form, not previously validated with other populations, were developed by principal investigators and statisticians and piloted with study staff. Surveys were password-protected; passwords were known only by study staff and were completed via a secure electronic data-capture system, REDCap. Before completing any questionnaires, both parents and adolescents were informed of the length of the time of the surveys, where data would be stored (ie, REDCap), the names of the principal investigators, and the purpose of the questionnaires. As all surveys consisted of prespecified measures, randomization of questions and adaptive questionnaires were not used, and incomplete questionnaires were included in analysis.

Eligible participants were randomly assigned to IPT or CBT. Participants were randomized using a computerized number generator created by a statistician; only the statistician knew the sequence. Randomization was performed by the statistician only after eligibility was determined. Every consecutive block of 2‐7 girls at each location was randomized to IPT or CBT. Randomization continued until a full cohort (4‐7 adolescents per group arm) was created. Neither interventionists, by design, nor outcome assessors were blind to assignment; research staff performing phlebotomy were blinded.

All virtual groups were cofacilitated by a psychologist and a graduate student with clinical training; groups were facilitated via the Health Insurance Portability and Accountability Act–compliant, secure platform Zoom. Both facilitators conducted both the IPT and CBT groups. Reminder emails were sent 24 hours prior to each group. Sessions lasted 90 minutes and took place weekly after school hours for 12 weeks. Fidelity and adherence were monitored through weekly supervision meetings that included feedback on audio-recorded sessions from a clinical psychologist with IPT and CBT expertise. Both programs included one 90-minute and two 30-minute individual sessions at the beginning, middle, and end of the 12-week group program, respectively.

At the first individual session, a parent or guardian briefly joined (10‐15 minutes) to review virtual group participation guidelines and problem-solve potential engagement barriers (eg, access to a device with camera and microphone in a private space). All participants received a text message reminding them to attend group 24 hours prior. Adolescents in both arms were encouraged to apply skills learned in group sessions to anxiety-provoking situations between sessions via home practice exercises facilitated through mailed binders containing handouts. Before each session, adolescents completed a mood-monitoring form, which assessed suicidality and self-harm; if either was endorsed, adolescents met with a psychologist for assessment and planning for safety monitoring.

Study flow, including time to enroll the total target sample, was tracked following CONSORT (Consolidated Standards of Reporting Trials) guidelines for pilot studies [59]. Recruitment feasibility was operationalized as enrollment of the target sample size (n=40) within 12 months. The percentage of eligible youth who enrolled was also calculated. Retention was tracked at each follow-up interval. Benchmarks for retention feasibility were defined as ≥80% retention at posttreatment or 12-week follow-up and ≥70% retention at 1-year follow-up. Feasibility of the study protocol was assessed by research staff completion of study flow checklists for standardized operating procedures at each interval. A threshold of ≥90% adherence to standardized procedures was expected, with ≤5% missing data for surveys, interviews, body measurements, blood pressure, and blood collection among completers at each interval.

### Primary Aims

Primary study aims were recruitment and retention feasibility, study protocol or data collection feasibility, intervention fidelity, and program acceptability. Recruitment and retention feasibility were assessed through timely recruitment, attendance, and retention at 12-week and 1-year follow-up. Intervention fidelity was assessed via fidelity rating scales. Fidelity rating scales were created by 2 PhD-level mental or behavioral health clinician–researchers not involved in intervention delivery and/or supervision. The mental or behavioral health clinician–researchers met consistently with experts in IPT and CBT over 2 months to develop the fidelity measures. Fidelity rating scales were adapted from previously developed IPT and CBT program adherence measures provided by the study’s primary investigators and were developed to reflect session-focused tasks specified in IPT and CBT intervention manuals. Fidelity measures were pilot tested using randomly chosen audio-recorded IPT and CBT sessions. Interrater reliability during pilot testing ranged between 0.95 and 1 (intraclass correlation coefficient 0.98; 95% CI 0.95‐1.00).

Intervention fidelity was assessed through review of 20% of randomly selected audio-recorded IPT and CBT sessions by the 2 PhD-level mental or behavioral health clinician–researchers. In line with existing fidelity research [60], a benchmark of ≥80% adherence was set. The IPT fidelity checklist included items such as “Have ≥1 group member discuss an interpersonal situation from the previous week and have the group ask questions; facilitate communication analysis if indicated.” A CBT checklist example is: “Present a scenario where teens cope successfully with a problem and provide self-ratings.” Items were rated 1=Yes, 0.5=Partially, or 0=No, with percentage fidelity calculated as the average adherence across all items and sessions per arm. Interrater reliability for 6 corated sessions was excellent (intraclass correlation coefficient 0.96; 95% CI 0.92-0.98).

To assess intervention acceptability, at 12-week follow-up, adolescents completed an adapted Treatment Acceptability Questionnaire [61], which measures likeability and credibility of IPT and CBT on a Likert scale (0=Not at all to 4=Definitely). Acceptability was defined as an average score ≥3 on likeability or credibility ratings within each arm. Acceptability was also characterized by attendance; ≥80% attendance at ≥10 of 12 group sessions was anticipated in both arms.

### Secondary Aims

Secondary aims were to describe within-arm changes in anxiety symptoms, disinhibited eating (emotional eating and loss-of-control eating), BMI indices and fat mass percentage, and cardiometabolic health indicators including blood pressure, cholesterol, triglycerides, glucose, and hemoglobin A_1c_ (HbA_1c_). Anxiety and eating behaviors were collected through online survey self-assessments, apart from the Eating Disorder Examination–Overeating Section [62], which was collected via interview by trained research staff. The STAI-C [56] assessed eligibility (total score ≥32; range 20‐60) and anxiety symptom severity. The STAI-C [56] has been validated for screening anxiety in youth with chronic health conditions with good psychometric properties [63]. Anxiety was measured at baseline and 1-year follow-up. Given the relevance of social anxiety in adolescents with elevated BMI, participants also completed the Social Phobia and Anxiety Inventory for Children [64], a 26-item self-report assessing somatic, cognitive, and behavioral aspects of social anxiety. Higher total scores (range 0‐52) indicate more symptoms. The Social Phobia and Anxiety Inventory for Children [64] is validated in children aged 8‐17 years, with good psychometric properties [65] and was administered at baseline, 12-week, and 1-year follow-ups.

The Emotional Eating Scale Adapted for Children and Adolescents [66] is a 25-item self-report assessing the urge to eat in response to negative affect. Items are measured on a 5-point scale [66], with higher scores reflecting a greater reported desire to eat when experiencing negative mood states [66]. Scores (range 0‐100) are summed. The Emotional Eating Scale Adapted for Children and Adolescents demonstrates good internal consistency and validity [67]. Loss-of-control eating was assessed via the Eating Disorder Examination–Overeating Section [62] administered by trained staff. Frequency of loss-of-control episodes in the past month was recorded as total episodes characterized by subjective inability to control eating any amount of food (subjective and/or objective binge-eating episodes) [62].

Height was measured in triplicate with a stadiometer (Model HM 200P; Charder Electronic COLTD) [68] and averaged. Height and weight were used to calculate BMI indices [69]. Fasting weight was measured on a calibrated digital scale connected to a Bod Pod [70]. Body fat percentage was measured via air displacement plethysmography using Bod Pod in a fasted state with minimal clothing and a swim cap [70].

Fasting blood samples were collected to measure glucose, cholesterol, triglycerides, and HbA_1c_. Resting blood pressure was measured in triplicate with an automatic monitor (Model UA-789AC; LifeSource) and averaged. Blood samples were processed at the University of Colorado Anschutz Medical Campus Core Lab using standard procedures.

CONSORT study flow information and descriptive summary statistics were used to assess recruitment and retention feasibility, study protocol or data collection feasibility, intervention fidelity, and program acceptability. Group medians, interquartile ranges, and 95% CIs were reported at each measurement interval, including baseline, 12-week follow-up, and 1-year follow-up, to illustrate patterns within arms over time. These descriptive data include all participants who completed each specific time point, resulting in the possibility of different sample sizes at each time point. Multiple imputation was not used, given that it relies on patterns in the data and therefore may be noisy or unreliable given the small sample size per arm [71]. To describe changes in clinical health-related outcomes, change scores from baseline to 12-week follow-up and baseline to 1-year follow-up were examined in each arm (IPT and CBT) separately, and this analysis included participants who completed both time points needed for calculation of the change score (ie, baseline and 12-week; baseline and 1-year).

Given the pilot nature of the study, in which we were focused on the estimation of a signal, a paired-samples 2-tailed *t* test was used to compare with a nonzero benchmark. Paired-samples *t* tests were used for normally distributed outcomes, and Wilcoxon signed rank tests were used when outcomes deviated from normality. Cohen *d* was calculated to describe effect sizes for normally distributed outcomes, while *r* was used for nonnormal outcomes. To control for multiple comparisons, adjusted *P* values using the Benjamini-Hochberg false discovery rate are reported. Because behavior, anxiety, BMI indices, and cardiometabolic health indicators were each prespecified as dependent variables, we applied a family-wise false discovery rate, grouping 6 outcomes related to anxiety, 8 outcomes related to disinhibited eating, 8 outcomes related to BMI indices, and 36 outcomes related to cardiometabolic indicators. For acceptability ratings, Mann-Whitney *U* tests were used to test for potential group differences across domains of acceptability.

### Ethical Considerations

The use of human subjects in this research was reviewed and approved by the USU IRB (number USUHS.2020‐048). Adolescents and parents or guardians were provided with informed consent and assent documents by trained study staff at the outset of the first screening visit. Informed consent documents included information on the nature and purpose of the study, study procedures including at-home device wear and group interventions, confidentiality, study benefits including compensation, study risks, alternative options (eg, therapy), and contact information if concerns arose. Adolescents were compensated for participating in various aspects of the study; in total, they could earn up to US $917 if they completed all visits, at-home data collection, surveys, group sessions, and follow-up visits. All data collected were deidentified.

Study staff who administered surveys were trained by a psychologist in safety and risk assessment. Any participants who expressed active self-harm or suicidal ideation were referred to crisis numbers and provided with counseling referrals. Adverse events were monitored by the study principal investigators (blinded for review) and the site principal investigators in conjunction with oversight from an external safety officer and study physician (blinded for review). Adverse events were promptly reported to the principal investigators, and any serious adverse events were immediately reported to the USU IRB. The investigators prepared a quarterly report for the Data Safety Officer with enrollment, retention, adverse events, and deviations. Following recommended guidelines, serious and/or unanticipated adverse events were reported within 5 business days to the IRB and the Data Safety Officer. The safety officer reviewed the quarterly data and safety-monitoring reports and, if needed, recommended corrective action or reporting of out-of-range laboratory data to the participant and their parents or guardians.

## Results

### Participants

A total of 40 adolescents were enrolled and randomized within 7 months. Mean age, race or ethnicity, and weight status were comparable across IPT and CBT arms. Table 1 displays demographic characteristics, BMI status, and socioeconomic information by arm. Adolescents in IPT had greater baseline percentage adiposity than adolescents in CBT (38.3% vs 32.0%), with no other significant group differences at baseline in any other characteristic. There was a trend toward a group difference in the racial composition of each arm, with IPT having more White (70.0% vs 55.0%) and Black or African American (30.0% vs 10.0%) participants, and the CBT group having more Asian (5.0% vs 0.0%) participants, more participants reporting more than 1 race (20.0% vs 0.0%), and more participants who did not report their race (10.0% vs 0.0%). Most IPT participants (85.0%) and all CBT participants had started their menstrual cycle at baseline. Average age at menarche for participants enrolled in IPT was 11.3 (SD 1.3), which was similar to participants in CBT (mean 11.5, SD 0.83). Most participants in IPT and CBT, respectively, reported experiencing menstruation every 28 days (64.7% vs 75.0%).

**Table 1.** Participant baseline demographic, weight, and socioeconomic characteristics by arm. Parent education is the highest level of education of the parent responding to the questionnaire.

	IPT^a^ (n=20)	CBT^b^ (n=20)	*P* value
Demographic			
Age (years), mean (SD)	14.9 (1.8)	14.8 (1.8)	.90
Started menstrual cycle, % (n/N)	85 (17/20)	100 (20/20)	.23
Age at menarche (years), mean (SD)	11.3 (1.3)	11.5 (0.83)	.66
28-day cycle, % (n/N)	64.7 (11/17)	75 (15/20)	.72
Race, n (%)			
White	14 (70)	11 (55)	.05
Black or African American	6 (30)	2 (10)	
Asian	0 (0.0)	1 (5)	
More than 1 race	0 (0.0)	4 (20)	
Missing or chose not to report	0 (0.0)	2 (10)	
Ethnicity, n (%)			
Hispanic/Latinx	2 (10)	3 (15)	.63
Non-Hispanic or non-Latinx	18 (90)	17 (85)	
Weight and adiposity, n (%)			
Obesity, BMI ≥95th percentile	13 (65)	12 (60)	.52
Overweight, BMI 85th to 94th percentile	6 (30)	8 (40)	
Nonoverweight, 75th to 84th percentile	1 (5)	0 (0.0)	
Percent fat mass, mean (SD)	38.3 (10.5)	32.0 (6.3)	.03
Socioeconomic			
Parent education, n (%)			
Less than high school	0 (0.0)	0 (0.0)	.25
High school or GED^c^	0 (0.0)	1 (5)	
Partial college	4 (20)	1 (5)	
Associate’s degree	0 (0.0)	0 (0.0)	
Four-year college degree	13 (65)	10 (50)	
Advanced degree	3 (15)	6 (30)	
Missing or chose not to report	0 (0)	2 (10)	
Parent subjective social status, mean (SD)			
Relative to the United States	5.3 (1.1)	5.9 (1.7)	.16
Relative to one’s own community	5.3 (1.3)	5.7 (1.7)	.42

a IPT: interpersonal psychotherapy.

b CBT: cognitive behavioral therapy.

c GED: General Educational Development.

### Primary Aims

**Figure 1.**
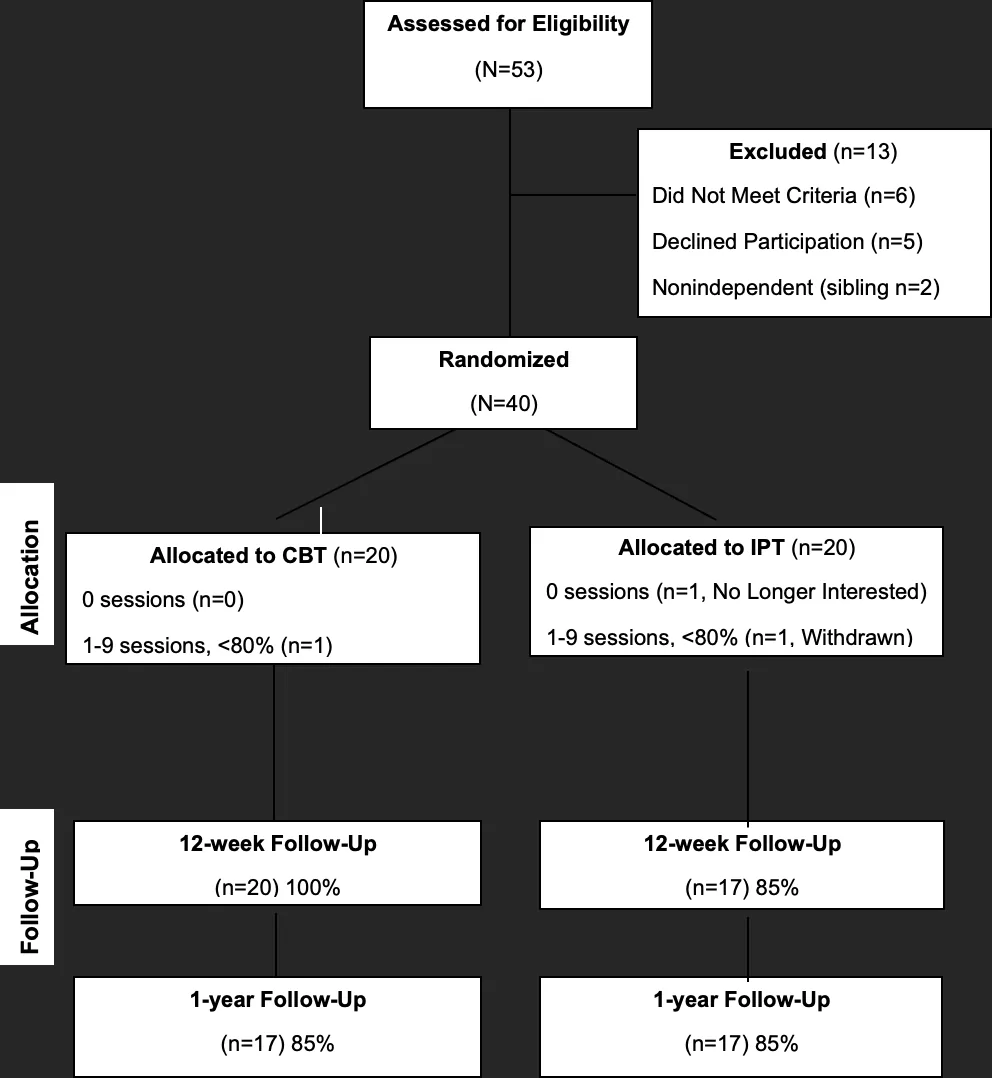
CONSORT (Consolidated Standards of Reporting Trials) study flow diagram for a web-based 2-site, 2-arm pilot and feasibility randomized controlled trial to improve cardiometabolic health in adolescent girls with elevated anxiety. CBT: cognitive behavioral therapy; IPT: interpersonal psychotherapy.

Recruitment occurred from February 2021 to August 2021, with the total target enrollment of 40 reached in 7 months, corresponding to a rate of 5‐6 enrolled participants per month across 2 sites. A total of 53 adolescents were assessed; 6 were ineligible. Of the 47 adolescents eligible after baseline screening, 40 (85.1%) chose to enroll. CONSORT study flow is depicted in Figure 1 (retention feasibility). Eighty-five percent (17/20) of the participants randomized to IPT completed the 12-week follow-up. In IPT, 1 participant withdrew before starting due to loss of interest, 1 IPT participant was withdrawn due to an unexpected, serious adverse event unrelated to the protocol, and 1 participant was unable to complete the 12-week follow-up due to scheduling conflicts. Overall, 100% (20/20) of those randomized to CBT completed the 12-week follow-up. At the 1-year follow-up, 85% (17/20) of the IPT participants and 85% (17/20) of the CBT participants completed follow-up.

Based on staff completion of standardized procedure checklists at each time point, compliance was 98.2% (7.86/8 procedures) at screening or baseline, 98.1% (7.85/8 procedures) at 12-week follow-up, and 96.6% (7.73/8 procedures) at 1-year follow-up. Table 2 presents data missingness by measurement type (questionnaires or interviews, body measurements, and phlebotomy) for each interval by arm. Expert ratings of intervention adherence indicated acceptable fidelity to IPT (mean 78%, SD 11%) and high fidelity to CBT (mean 94%, SD 9%).

**Table 2.** Missingness of data for adolescent participants who completed each interval within arm over time^a^.

	IPT^b^, % (n/N)			CBT^c^, % (n/N)		
	Baseline (n=20)	12 weeks (n=17)	1 year (n=17)	Baseline (n=20)	12 weeks (n=20)	1 year (n=17)
Questionnaire						
Treatment acceptability	N/A^d^	5.9 (1/17)	N/A	N/A	10.0 (2/20)	N/A
Anxiety	0.0 (0/20)	N/A	0.0 (0/17)	0.0 (0/20)	N/A	0.0 (0/17)
Social anxiety	5 (1/20)	5.9 (1/17)	0.0 (0/17)	5 (1/20)	15 (3/20)	0.0 (0/17)
Emotional eating	0.0 (0/20)	5.9 (1/17)	0.0 (0/17)	0.0 (0/20)	10 (2/20)	0.0 (0/17)
Interview						
Loss-of-control eating	0.0 (0/20)	0.0 (0/17)	5.9 (1/17)	0.0 (0/20)	5 (1/20)	5.9 (1/17)
Body measures						
BMI indices	0.0 (0/20)	0.0 (0/17)	0.0 (0/17)	0.0 (0/20)	0.0 (0/20)	0.0 (0/17)
Body fat^e^	0.0 (0/20)	0.0 (0/17)	0.0 (0/17)	5 (1/20)	5 (1/20)	0.0 (0/17)
SBP/DBP^f^	5 (1/20)	5.9 (1/17)	0.0 (0/17)	0.0 (0/20)	0.0 (0/20)	0.0 (0/17)
Phlebotomy						
Lipids^g^	20 (4/20)	17.6 (3/17)	23.5 (4/17)	15 (3/20)	20 (4/20)	23.5 (4/17)
HbA_1c_^h^	25 (5/20)	17.6 (3/17)	11.8 (2/17)	20 (4/20)	30 (6/20)	17.6 (3/17)
Glucose	20 (4/20)	17.6 (3/17)	23.5 (4/17)	15 (3/20)	20 (4/20)	23.5 (4/17)

a Values are % missing (missing data/n completed interval).

b IPT: interpersonal psychotherapy.

c CBT: cognitive behavioral therapy.

d N/A: not applicable because not collected at this interval.

e Body fat: air displacement plethysmography/Bod Pod.

f SBP/DBP: systolic and diastolic blood pressure.

g Lipids: triglycerides and cholesterol.

h HbA_1c_: hemoglobin A_1c_.

**Figure 2.**
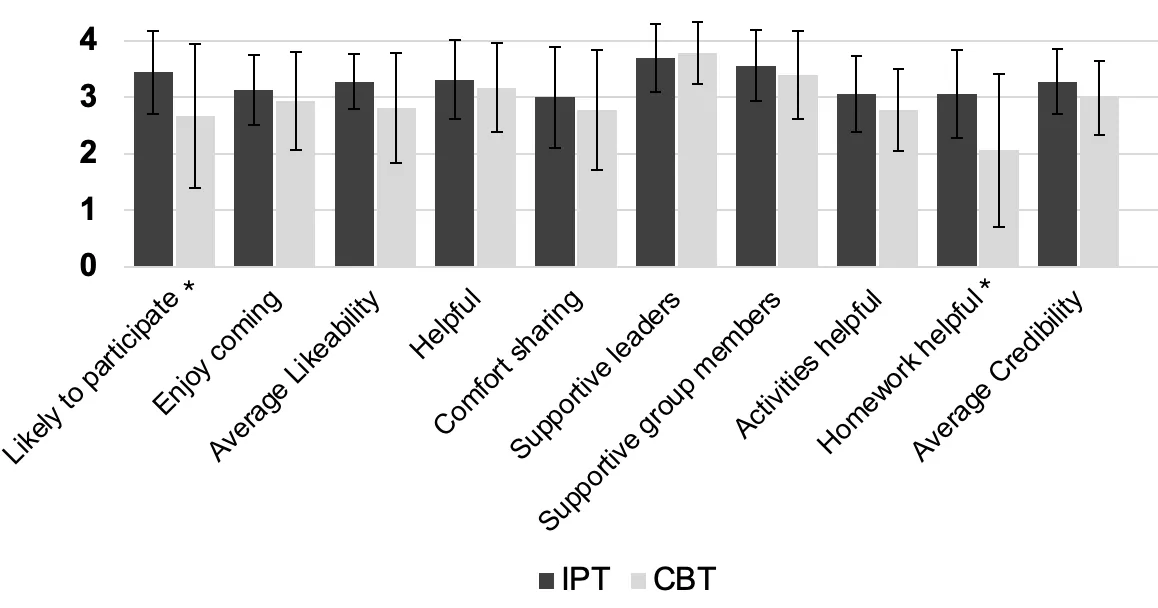
Average acceptability and credibility ratings by intervention arm for a web-based 2-site, 2-arm pilot and feasibility randomized controlled trial to improve cardiometabolic health in adolescent girls with elevated anxiety. Acceptability ratings among adolescent girls with elevated anxiety and above-average weight randomized to 12-week, virtual group IPT or 12-week, virtual group CBT. Values are mean (SD) on 0-4 scale, with 0=Lowest likability or credibility and 4=Highest likability or credibility. *Statistically significant and reported in the aforementioned text. The values for likely to participate are as follows: CBT: mean 2.67 (SD 1.28); IPT: mean 3.44 (SD 0.18); and *t* test: *t*_32_=−2.12; *P*=.04. The values for homework helpful are as follows: CBT: mean 2.17 (SD 1.34); IPT: mean 3.13 (SD 0.81); and *t* test: *t*_32_=−2.45; *P*=.01. CBT: cognitive behavioral therapy; IPT: interpersonal psychotherapy.

Figure 2 shows average acceptability ratings by arm. Acceptability (likability and credibility) survey results exclude participants with missing survey data, resulting in 16 adolescents completing surveys in IPT and 18 in CBT. Nonparametric tests of group differences revealed higher ratings of the helpfulness of at-home activities in the IPT group (*U*=204.00, mean rank=21.25 in IPT vs 14.17 in CBT; *P*=.04). There was also a trend where IPT participants were more likely to report that they would participate in the program if they could do it over (*U*=193.50, mean rank 20.59 vs 14.75 in CBT; *P*=.09). All Mann-Whitney comparisons should be interpreted with caution, however, given the small sample size.

Descriptively, in IPT, 87.5% (14/16) of adolescents reported that they would “mostly” or “definitely” complete the program again (3 or 4 on a 0‐4 scale), 87.5% (14/16) found the group “very” to “definitely” enjoyable, and 87.5% (14/16) thought the group would be “mostly” or “extremely” helpful for peers. Most adolescents in IPT felt “very” or “extremely” comfortable sharing and asking questions (12/16, 75%), viewed facilitators (15/16, 93.8%) and group members (15/16, 93.8%) as supportive, found group activities “very” or “extremely” helpful (13/16, 81.3%), and saw home practice exercises as “very” or “extremely” useful (12/16, 75%).

In CBT, most adolescents (11/18, 61.1%) reported that they would “mostly” or “definitely” complete the program again, 66.7% (12/18) found the group “very” to “definitely” enjoyable, and 72.2% (13/18) thought the group would be “mostly” or “extremely” helpful for their peers. Many adolescents also felt comfortable sharing and asking questions (13/18, 72.2%). Similar to IPT, adolescents viewed facilitators (17/18, 94.4%) and group members (17/18, 94.4%) as supportive. Most adolescents in CBT found group activities “very” or “extremely” helpful (13/18, 72.2%), but a minority saw home practice exercises as “very” or “extremely” useful (8/18, 44.4%).

In the intent-to-treat sample, 90% (18/20) in IPT and 95% (19/20) in CBT received ≥80% of the intervention dose (≥10 of 12 group sessions). Median attendance was 11 sessions in IPT and 12 in CBT. One IPT participant withdrew before starting due to loss of interest. One IPT participant was withdrawn due to an unexpected, serious adverse event unrelated to the protocol; the participant reported worsening mental health symptoms that necessitated hospitalization and precluded ongoing research participation. Excluding withdrawn participants, mean attendance was 11.00 (SD 1.46) sessions in IPT and 11.55 (SD 1.36) sessions in CBT.

### Secondary Aims

Key clinical outcomes at each interval by arm are detailed in Table 3. Change scores in anxiety, disinhibited eating, BMI indices, and adiposity, as well as cardiometabolic health indices adjusted for multiple comparisons and within-arm effect sizes, are shown in Table 4. From baseline to 12-week follow-up, adolescents enrolled in CBT (but not IPT) showed improvement in emotional eating and adolescents enrolled in IPT (but not CBT) demonstrated improvement in BMI percentile. From baseline to 1-year follow-up, adolescents enrolled in IPT (but not CBT) showed improvement in general anxiety, social anxiety, and HbA_1c_, while adolescents in CBT (but not IPT) demonstrated improvements in emotional eating. Both arms demonstrated significant improvement in BMI percentile at 1-year follow-up. No significant changes were observed in loss-of-control eating, BMI, fat mass percentage, blood pressure, triglycerides, total cholesterol, high-density lipoprotein cholesterol, low-density lipoprotein cholesterol, or glucose at 1-year follow-up in either arm.

**Table 3.** Anxiety, emotional eating, BMI or adiposity, and cardiometabolic health indices by intervention arm across baseline, 12-week, and 1-year intervals. Denominators reflect participants who completed assessments at each interval.

	IPT^a^						CBT^b^					
	Baseline		12 weeks		1 year		Baseline		12 weeks		1 year	
Measure	n	Median (IQR)	n	Median (IQR)	n	Median (IQR)	n	Median (IQR)	n	Median (IQR)	n	Median (IQR)
Anxiety and disinhibited eating												
Anxiety	20	41.5 (35.3-46.0)	—^c^	NA^d^	17	*35.0 *(*29.0-42.0*^e^)	20	39.5 (35.5-43.8)	—	NA	17	38.0 (30.5-42.0)
Social anxiety	19	24.0 (16.0-35.0)	16	17.50 (14.0-25.0)	17	*17.0 *(*9.5-24.5*)	19	23.0 (15.0-32.0)	17	23.0 (12.0-28.5)	17	11.0 (4.0-25.0)
Emotional eating	20	1.5 (1.1-1.9)	16	1.3 (0.7-1.7)	17	1.2 (0.5-1.6)	20	1.3 (1.0-2.1)	18	*0.3 *(*0.1-0.8*)	17	*0.5 *(*0.2-1.1*)
Loss of control	20	0.0 (0-3)	17	0.0 (0-1)	16	0.0 (0-0.75)	20	0.0 (0-0)	19	0.0 (0-0)	16	0.0 (0-0)
BMI and adiposity												
BMI, kg/m^2^	20	31.4 (25.6-37.7)	17	32.0 (25.6-38.8)	17	32.6 (24.4-37.9)	20	28.2 (26.0-31.0)	20	28.1 (26.6-31.3)	17	30.0 (25.9-32.2)
BMI, percentile	20	97.0 (91.0-99.0)	17	*98.0*^f^ (*89.5-99.0*)	17	*98.0 *(*84.5-99.0*)	20	95.0 (93.0-97.8)	20	95.0 (94.0-97.0)	17	*95.0 *(*90.0-98.0*)
Fat mass, percentage	20	39.4 (28.3-46.6)	17	40.6 (30.0-43.7)	17	41.0 (30.9-47.7)	19	30.8 (27.0-37.5)	19	30.0 (27.8-32.3)	17	32.1 (27.2-36.6)
Cardiometabolic health												
SBP^g^, mm Hg	19	111.3 (108.7-122.7)	16	114.5 (106.8-125.2)	17	111.0 (103.3-126.3)	20	119.8 (111.3-126.0)	20	123.5 (110.8-126.0)	17	111.3 (108.2-124.3)
DBP^h^, mm Hg	19	78.7 (69.3-84.7)	16	76.5 (72.3-83.7)	17	67.7 (64.7-77.6)	20	76.0 (70.1-83.8)	20	79.7 (75.0-83.8)	17	72.0 (63.2-84.0)
Triglycerides, mg/dL	16	71.5 (59.0-97.0)	14	67.5 (51.8-96.5)	13	52.0 (44.5-96.0)	17	103.0 (69.0-128.0)	16	97.0 (68.5-152.5)	13	84.0 (70.5-119.5)
Total cholesterol, mg/dL	16	155.5 (138.0-165.3)	14	151.5 (138.0-162.8)	13	143.0 (126.5-159.5)	17	151.0 (125.0-165.5)	16	152.5 (130.3-166.8)	13	144.0 (124.5-174.5)
HDL^i^ cholesterol, mg/dL	16	46.0 (39.0-49.0)	14	45.5 (38.5-49.3)	13	41.0 (38.00-53.5)	17	41.0 (36.5-49.0)	16	45.0 (40.8-50.8)	13	42.0 (35.5-46.0)
LDL^j^ cholesterol, mg/dL	16	104.0 (87.3-117.3)	14	98.0 (88.0-113.0)	13	94.0 (75.5-105.5)	17	109.0 (74.0-116.0)	16	94.0 (75.0-114.5)	13	94.0 (72.5-118.0)
HbA_1c_^k^	15	5.6 (5.4-5.7)	14	5.6 (5.4-5.7)	15	*5.2* (*5.1-5.5*)	16	5.5 (5.3-5.8)	16	5.7 (5.4-5.9)	14	5.3 (5.0-5.5)
Glucose, mg/dL	16	84.5 (78.3-90.8)	14	83.5 (77.8-87.0)	13	83.0 (80.0-92.0)	17	83.0 (81.0-85.0)	16	85.0 (81.5-88.0)	13	85.0 (83.5-89.0)

a IPT: interpersonal psychotherapy.

b CBT: interpersonal psychotherapy.

c Not available because not collected at this interval.

d N/A: not applicable because not collected at this interval.

e Values in italics are statistically significant.

f Time point specific summary statistics in Table 3 are based upon complete data (n=20, 17 at baseline and 12 weeks, respectively), whereas estimated difference scores in Table 4 hinge on complete information across both time points (n=17).

g SBP: systolic blood pressure.

h DBP: diastolic blood pressure.

i HDL: high-density lipoprotein.

j LDL: low-density lipoprotein.

k HbA_1c_: hemoglobin A_1c_.

**Table 4.** Within-condition changes and effect sizes within interpersonal psychotherapy and cognitive behavioral therapy arms using complete case analysis^a^. As this is a pilot study that does not formally compare groups, the authors caution against comparing treatments.

Characteristics and arms	n	*t* test (*df*) /*Z*^b^^,^^c^	*P* value	FDR^d^-adjusted *P* value	Mean/median ∆^e^	95% CI ∆	*d*/*r^f^*^,^^g^ effect size
Anxiety							
∆ Baseline to 1 year							
IPT^h^	17	−2.91 (16)	.01	.03	−5.18	−8.95 to −1.40	−0.71
CBT^i^	17	−2.29 (16)	.04	.08	−3.76	−7.24 to −0.28	−0.56
Social anxiety							
∆ Baseline to 12 weeks							
IPT	15	−1.94 (14)	.07	.08	−5.53	−11.66 to 0.60	−0.50
CBT	16	−1.47 (15)	.16	.16	−2.38	−5.83 to 1.08	−0.37
∆ Baseline to 1 year							
IPT	16	−2.90 (15)	.01	.03	−7.13	−12.37 to −1.88	−0.72
CBT	16	−2.14 (15)	.05	.08	−6.75	−13.47 to −0.03	−0.54
Emotional eating							
∆ Baseline to 12 weeks							
IPT	16	−2.25 (15)	.04	.08	−0.25	−0.48 to −0.01	−0.56
CBT	18	−4.45 (17)	<.001	.01	−0.82	−1.21 to −0.43	−1.05
∆ Baseline to 1 year							
IPT	17	−1.89 (16)	.08	.13	−0.25	−0.54 to 0.03	−0.46
CBT	17	−2.88 (16)	.01	.04	−0.68	−1.18 to −0.18	−0.70
Loss-of-control eating							
∆ Baseline to 12 weeks							
IPT	17	−1.47	.14	.19	0.00	N/A^j^	−0.52
CBT	19	0	≥.99	>.99	0.00	N/A	0.00
∆ Baseline to 1 year							
IPT	16	−2.09	.04	.08	0.00	N/A	−0.70
CBT	16	−0.82	.41	.46	0.00	N/A	−0.47
BMI (kg/m^2^)							
∆ Baseline to 12 weeks							
IPT	17	−0.55 (16)	.59	.76	−0.18	−0.86 to 0.51	−0.13
CBT	20	0.36 (19)	.72	.76	0.09	−0.40 to 0.57	0.08
∆ Baseline to 1 year							
IPT	17	−0.31 (16)	.76	.76	−0.16	−1.28 to 0.95	−0.08
CBT	17	0.69 (16)	.50	.76	0.25	−0.53 to 1.03	0.17
BMI percentile							
∆ Baseline to 12 weeks							
IPT	17	−2.53	.01	.04	−1.00^e^	N/A	−0.80
CBT	20	−1.24	.22	.44	0.00	N/A	−0.39
∆ Baseline to 1 year							
IPT	17	−2.85	.004	.03	−1.00	N/A	−0.82
CBT	17	−2.29	.02	.05	−1.00	N/A	−0.63
Fat mass (%)							
∆ Baseline to 12 weeks							
IPT	17	−1.60 (16)	.13	.64	−2.09	−4.86 to .68	−0.39
CBT	19	−0.94 (18)	.36	.78	−1.52	−4.94 to 1.90	−0.21
∆ Baseline to 1 year							
IPT	17	1.38 (16)	.19	.64	1.50	−0.80 to 3.78	0.34
CBT	16	−0.20 (15)	.84	.98	−0.25	−2.93 to 2.42	−0.05
SBP^k^ (mm Hg)							
∆ Baseline to 12 weeks							
IPT	16	−0.70 (15)	.49	.83	4.00	N/A	−0.18
CBT	20	−0.90 (19)	.37	.78	0.17	N/A	−0.20
∆ Baseline to 1 year							
IPT	16	−0.65 (15)	.53	.83	−2.23	−9.56 to 5.10	−0.16
CBT	17	−1.26 (16)	.23	.64	−3.37	−9.11 to 2.37	−0.30
DBP^l^ (mm Hg)							
∆ Baseline to 12 weeks							
IPT	16	−0.93 (15)	.37	.78	−5.88	−19.28 to 7.52	−0.23
CBT	20	0.65 (19)	.52	.83	1.55	−3.44 to 6.54	0.15
∆ Baseline to 1 year							
IPT	16	−2.77 (15)	.01	.12	−11.00	−19.48 to −2.52	−0.69
CBT	17	−1.28 (16)	.22	.64	−3.67	−9.73 to 2.40	−0.31
Triglycerides (mg/dL)							
∆ Baseline to 12 weeks							
IPT	13	−0.13 (12)	.90	.98	−0.62	−10.60 to 9.36	−0.04
CBT	14	1.53 (13)	.15	.64	11.00	−4.52 to 26.52	0.41
∆ Baseline to 1 year							
IPT	12	−1.33 (11)	.21	.64	−8.75	−23.23 to 5.73	−0.38
CBT	11	−0.70 (10)	.50	.83	−6.09	−25.40 to 13.21	−0.21
Total cholesterol (mg/dL)							
∆ Baseline to 12 weeks							
IPT	13	−0.45 (12)	.66	.92	−1.23	−7.19 to 4.73	−0.13
CBT	14	−0.37 (13)	.72	.92	−0.93	−6.30 to 4.45	−0.10
∆ Baseline to 1 year							
IPT	12	−2.31 (11)	.04	.29	−10.50	−20.53 to −0.47	−0.67
CBT	11	−1.07 (10)	.31	.78	−5.64	−17.35 to 6.08	−0.32
HDL^m^ (mg/dL)							
∆ Baseline to 12 weeks							
IPT	13	0.00 (12)	≥.99	>.99	0.00	−2.47 to 2.47	0.00
CBT	14	−0.33 (13)	.75	.92	−0.36	−2.73 to 2.01	−0.09
∆ Baseline to 1 year							
IPT	12	0.77 (11)	.46	.83	0.92	−1.69 to 3.52	0.22
CBT	11	0.00 (10)	≥.99	>.99	0.00	−3.69 to 3.69	0.00
LDL^n^ (mg/dL)							
∆ Baseline to 12 weeks							
IPT	13	0.15 (12)	.88	.98	0.31	−4.16 to 4.77	0.04
CBT	14	−0.33 (13)	.75	.92	−0.86	−6.51 to 4.79	−0.09
∆ Baseline to 1 year							
IPT	12	−1.73 (11)	.11	.64	−9.50	−21.59 to 2.59	−0.50
CBT	11	−1.32 (10)	.22	.64	−5.27	−14.17 to 3.62	−0.40
HbA_1c_^o^							
∆ Baseline to 12 weeks							
IPT	12	0.59 (11)	.57	.86	0.03	−0.09 to 0.16	0.17
CBT	13	2.29 (12)	.04	.29	0.12	0.01 to 0.23	0.63
∆ Baseline to 1 year							
IPT	12	−5.75 (11)	.001	.04	−0.35	−0.48 to −0.23	−1.66
CBT	11	−3.02 (10)	.01	.12	−0.23	−0.39 to −0.06	−0.91
Fasting glucose (mg/dL)							
∆ Baseline to 12 weeks							
IPT	13	0.30 (12)	.77	.92	0.54	−3.32 to 4.40	0.08
CBT	14	−0.39 (13)	.70	.92	−0.64	−4.18 to 2.89	−0.11
∆ Baseline to 1 year							
IPT	12	0.65 (11)	.53	.83	1.00	−2.37 to 4.37	0.19
CBT	11	0.09 (10)	.93	.98	0.18	−4.26 to 4.62	0.03

a Values reported in the mean/median Δ column are descriptive; Wilcoxon signed-rank tests evaluate median change.

b *t*: *t* test,

c *Z*: Wilcoxon signed rank test,

d FDR: false discovery rate.

e Time point specific summary statistics in Table 3 are based upon complete data (n=20, 17 at baseline and 12 weeks, respectively), whereas estimated difference score in Table 4 hinges on complete information across both time points (n=17).

f *d*: effect size for *t* tests.

g *r*: effect size for Wilcoxon signed rank tests.

h IPT: interpersonal psychotherapy..

i CBT: cognitive behavioral therapy.

j N/A: not applicable because measure not collected at this interval.

k SBP: systolic blood pressure.

l DBP: diastolic blood pressure.

m HDL: high-density lipoprotein.

n LDL: low-density lipoprotein.

o HbA_1c_: hemoglobin A_1c_.

## Discussion

### Principal Findings

This study was the first to directly pilot the feasibility, acceptability, and exploratory outcomes on anxiety and cardiometabolic health markers of web-based, group IPT and CBT for adolescent girls with above-average weight and elevated anxiety symptoms. The target sample size of 40 adolescents was achieved across 2 sites in 7 months, with an enrollment rate of 5‐6 participants per month. Of eligible adolescents, 85% (40/47) elected to enroll. Total retention was 93% (37/40) at 12-week follow-up and 85% (34/40) at 1-year follow-up. Specifically, at 12-week follow-up, 95% (19/20) of participants from USU and 90% (18/20) of participants at CSU completed the posttreatment visit (*χ*^2^_2_=1.3; *P*=.55). At both sites, 85% (17/20) of participants completed 1-year follow up (*χ*^2^_1_=0.00; *P*>.99). Protocol adherence by research staff was high (>96%) across time points. Data missingness ranged from 0% to 30%, with no missing height or weight data. Questionnaire missingness ranged from 0% to 15%, and interview and body fat measurements missingness never exceeded 5.9%. Cardiometabolic measures requiring phlebotomy had the greatest missingness (15%‐25% at baseline, 11.8%-30% at follow-up). Intervention fidelity ratings indicated high CBT adherence (>90%); IPT adherence was just below the 80% threshold. Overall acceptability was high, with >90% (n≥17) receiving at least 80% of intervention dosage in both arms. Home practice assignments were rated as the least helpful aspect of both IPT and CBT web-based group interventions.

### Principal Findings in the Context of Prior Work

Recruitment success likely reflects multifaceted strategies (letters, flyers, social media, and listserves) and the offer of evidence-based mental health interventions in both arms rather than a nonactive comparator, as reflected in qualitative work on research recruitment with adolescents [72] and similar to other trials comparing 2 evidence-based interventions [73]. Furthermore, the inclusion criteria of elevated anxiety symptoms and BMI ≥75th percentile, rather than overweight (≥85th percentile) or obesity (≥95th percentile), were appealing as they emphasized anxiety and stress prevention rather than weight loss, consistent with prior qualitative work [74]. Previous research indicates that effective retention strategies include flexible scheduling, easy interactions with research staff, and frequent contact [75]. These strategies were reflected in this trial, as staff offered flexible scheduling and focused on ongoing engagement via calls, birthday cards, and newsletters.

Few studies have evaluated therapist adherence to IPT and CBT simultaneously. Ekeblad and colleagues [44] compared IPT and CBT fidelity using the Collaborative Study Psychotherapy Rating Scale—6 [76]. Adherence was found to be at or above 80% [45]. The Collaborative Study Psychotherapy Rating Scale—6 was designed to assess IPT with *adult individuals* rather than *groups of adolescents,* preventing its use in this study. However, the adherence found in both studies is similar to the fidelity findings in the current protocol. We speculate that CBT’s greater structure likely supported higher adherence, whereas IPT’s flexibility in interpersonal content demands facilitator spontaneity and responsiveness to adolescent affect. The most missed IPT component included session summaries in favor of increased time in role-plays, highlighting training needs to balance fidelity and therapist competence. Competence in the context of fidelity refers to the idea that therapists can draw on a wide range of interpersonal and emotional skills to address challenges and respond to participants [77]. Below desired (ie, 78% rather than 80%) IPT fidelity ratings may reflect a greater emphasis on evaluation using a structured protocol rather than therapist competence and responsiveness to adolescent needs in IPT. Notably, adolescents reported greater enjoyment of IPT, potentially reflecting its personalized nature. Both IPT and CBT were led by the same facilitators, suggesting that this effect is not attributable to facilitator differences. The current results support the importance of measuring competence and personalizing treatment [78].

Despite adolescents’ report that homework was the least helpful aspect of both groups, homework predicts clinical improvement [79]. Lower perceived helpfulness of homework may relate to negative attitudes toward academic homework and the burden of therapeutic tasks [80]. Additionally, adolescents diagnosed with anxiety may struggle to complete homework due to forgetfulness or misunderstandings of the most effective way to implement skills [81].

Overall, data missingness appears similar to other clinical trials involving phlebotomy with adolescents [82,83]. Despite this, virtual delivery of both group programs was successful. Virtual group formats are convenient for busy adolescents, minimizing travel burden and fostering peer connection [84]. Adolescents rated groups as enjoyable, comfortable, and helpful, with facilitators and peers perceived as supportive, similar to other group-based formats for adolescents [85].

### Secondary Findings

Moderate to large reductions in general and social anxiety were observed at 1-year follow-up for adolescents enrolled in IPT, with no significant social anxiety changes at 12-week or postintervention follow-up. This pattern may indicate that treatment effects develop over time. Immediately post intervention, adolescents enrolled in IPT demonstrated significant reductions in BMI percentile, as well as at 1-year follow-up. Additionally, adolescents enrolled in CBT experienced significant change in both emotional eating and BMI percentile at 1 year postintervention follow-up. These preliminary and exploratory findings provide an initial signal that IPT and CBT may influence mechanisms that trigger disinhibited eating, yet this hypothesis would need to be explicitly tested in larger samples powered to study mechanistic effects. The positive pattern of change in eating behavior in CBT, although highly preliminary, is notable in the context of prior data that disinhibited eating often worsens, and behavioral weight interventions typically show rebound effects posttreatment [86]. Some metabolic outcomes improved descriptively, with CBT showing improvements in BMI percentile and IPT demonstrating HbA_1c_ improvements at 1 year. While highly preliminary, these initial data suggest that web-based group anxiety interventions have the potential to support healthier cardiometabolic trajectories in at-risk adolescent girls.

### Secondary Findings in Context of Prior Work

Consistent with previous literature [87], both IPT and CBT resulted in moderate to large reductions in anxiety symptoms. Extant research also supports the use of mental health interventions to address the potential mediators of physical health conditions [37]. For example, mental health interventions have been found to improve both anxiety symptoms and cardiometabolic health in adolescents with excess weight [86].

### Strengths and Limitations

Strengths of this study include methodological rigor with strong recruitment, retention, protocol adherence, intervention attendance, and acceptability. Limitations include the following.

First, this study has limited power. Notably, the study was not powered to assess comparative effects; within-arm changes should be interpreted cautiously, given the small sample size. Likewise, as this study was a pilot of 2 active interventions without an inactive control arm, any within-arm improvements cannot be attributed to the specific modalities tested; it is possible that changes were influenced by natural maturation, nonspecific therapeutic attention effects, and/or regression to the mean.

Second, missing data were not uniform across outcomes and were concentrated in phlebotomy-derived cardiometabolic measures. Whereas questionnaire, interview, and anthropometric outcomes were largely complete, blood-based measures had 15%‐30% missingness across arms and time points, exceeding the study’s a priori feasibility benchmark. Accordingly, exploratory findings for metabolic (eg, HbA_1c_) outcomes should be interpreted cautiously, as missingness likely reduced precision, effective sample size, and power to detect small to-moderate effects. Because this pilot was not designed or powered to formally evaluate missing data mechanisms, the extent of potential bias remains uncertain. Exploratory comparisons of participants with versus without follow-up phlebotomy data on baseline characteristics showed no meaningful baseline differences, suggesting limited evidence of systematic missingness based on observed variables. However, the group of participants with missing baseline phlebotomy data had higher baseline BMI percentile (mean 97.70, SD 0.64 vs mean 94.12, SD 4.90; *P*=.01) and a greater proportion of Hispanic and Latinx participants (40% vs 6%, Fisher exact test *P*=.03) versus the group with baseline phlebotomy data. Therefore, we cannot rule out the possibility of systematic missingness at baseline based on higher BMI percentile and ethnicity. In future studies, it will be important to ensure that baseline measures of phlebotomy are collected across BMI strata and race and ethnicity, particularly in youth with higher BMI and Hispanic Latinx ethnicity, addressing any potential barriers in youth with more severe obesity or Hispanic or Latinx ethnicity.

Third, although IPT intervention fidelity did not reach prespecified thresholds, most of the feasibility and acceptability markers did, suggesting strong support for feasibility in 7 of 9 dimensions with room to improve in both phlebotomy and IPT fidelity. As IPT was just below the 80% benchmark and we did not measure competence, it is possible that the therapeutic dosage was suboptimal. Nevertheless, the most common missing fidelity requirement was summarizing the session, suggesting that core content was administered to fidelity.

Fourth, while social anxiety symptoms were assessed at baseline and all follow-up intervals, general anxiety symptoms were assessed only at baseline and 1 year as part of an effort to limit participant questionnaire fatigue and given the study’s focus on feasibility and acceptability as primary outcomes. Additional limitations include that homework completion was not objectively measured.

Fifth, participants could not be blinded, which increased the risk for type I error due to the multiplicity of outcomes. Sixth, the absence of qualitative data limits participant perspective on intervention experience. Seventh, the sample was demographically homogenous, thereby limiting external validity and generalizability of the feasibility and acceptability findings to lower-income or minority populations. Eighth, reductions in loss-of-control eating episodes must be considered cautiously, as not all adolescents had loss of control at baseline and frequencies of loss-of-control episodes were low. Finally, there was a group difference in percentage adiposity at baseline, with the IPT arm having higher percentage adiposity than the CBT arm; it is possible that outcomes pertaining both to adiposity and other variables may relate to this baseline characteristic.

### Future Work

Future fully powered trials should be designed to test comparative efficacy between IPT and CBT, using a prespecified primary end point and adequate sample size. In the present pilot, within-arm effect sizes for key outcomes were generally in the moderate to large range. For planning purposes, anchoring to a moderate effect (*d*=0.50; α=.05, 80% power), a future trial would require approximately 64 participants per arm with complete data, corresponding to approximately 93‐99 participants per arm after accounting for approximately 20%‐30% attrition and biospecimen missingness. Larger samples would be required to adequately detect between-group differences in treatment response. Furthermore, future research should engage adolescent interest holders to identify barriers and facilitators to homework adherence and explore digital or technological supports as enhancement to web-based group interventions.

Therefore, areas of refinement include phlebotomy procedures, specifically, planning for larger sample sizes to accommodate biospecimen missingness and prospectively documenting reasons for missed or unsuccessful blood draws, IPT training or supervision related to adherence and competence, sufficient retention to confirm the impact of IPT versus CBT on cardiometabolic health for adolescent girls, and including nonactive control groups. Specifically, future trials should anticipate missing data from phlebotomy and consider specialized personnel or noninvasive alternatives (eg, ultrasound) to reduce missingness; use of finger pricks or nonfasting measures would also be expected to improve data completeness [83]. Fully powered efficacy trials should also aim to assess the mediational pathways of the mechanisms of both IPT and CBT, evaluate generalized anxiety at all follow-up intervals to better characterize trajectories of anxiety, plan for greater missingness in power estimations, and account for pubertal changes during the follow-up period. Additionally, future trials should test the impact of interventions for girls with more frequent loss-of-control eating and/or binge eating disorder.

### Conclusions

This pilot trial provides initial support for feasibility and acceptability of virtual group IPT and CBT for adolescent girls with anxiety symptoms who also are at risk for future, preventable cardiometabolic disease due to above-average BMI. Specifically, 7 out of 9 a priori benchmarks demonstrated feasibility (ie, recruitment, retention, protocol adherence, nonphlebotomy data missingness, CBT intervention fidelity, and both IPT and CBT intervention likability and credibility). The results also pointed to 2 areas to refine (ie, phlebotomy missingness and IPT intervention fidelity) for a future trial.

Given the high prevalence of anxiety among adolescent girls with excess weight and evidence that mental health interventions have the potential to improve not only mental health but also behavioral and cardiometabolic outcomes, targeting anxiety represents a promising and novel prevention strategy. Routine pediatric clinical care, particularly care designed to address excess weight and its comorbidities (eg, lifestyle medicine), would likely benefit from integrated group mental health interventions to address mental health concerns such as anxiety. Web-based group interventions may offer a scalable and accessible approach to addressing these intersecting risks and will benefit from continued interdisciplinary collaboration across psychology, pediatrics, and cardiometabolic health. Yet, the current results must be considered preliminary. Building on the feasibility and acceptability demonstrated in this pilot, a fully powered randomized controlled trial is warranted to evaluate the efficacy and mechanisms of virtual group IPT and CBT in this population.

## Supplementary material

10.2196/82781 Checklist 1 CONSORT-EHEALTH checklist (V 1.6.1).

## Abbreviations

CBT: cognitive behavioral therapy
CONSORT: Consolidated Standards of Reporting Trials
CSU: Colorado State University
HbA_1c_: hemoglobin A_1c_
IPT: interpersonal psychotherapy
IRB: institutional review board
STAI-C: State-Trait Anxiety Inventory for Children
USU: Uniformed Services University
