# Supplementary material for: Improving Cardiometabolic Health in Adolescent Girls With Elevated Anxiety: Web-Based Multisite, Pilot, and Feasibility Randomized Controlled Trial
Source: JMIR Cardio. 2026 Sep 17;10:e82781. doi: 10.2196/82781 (PMC13585365; doi:10.2196/82781)
Supplement: Supplementary file 1 [file cardio-v10-e82781-s001.pdf]

CONSORT-EHEALTH (V 1.6.1) - Submission/Publication Form

The CONSORT-EHEALTH checklist is intended for authors of randomized trials evaluating web-based and Internet-based applications/interventions, including mobile interventions, electronic games (incl multiplayer games), social media, certain telehealth applications, and other interactive and/or networked electronic applications. Some of the items (e.g. all subitems under item 5 - description of the intervention) may also be applicable for other study designs.

The goal of the CONSORT EHEALTH checklist and guideline is to be
a) a guide for reporting for authors of RCTs,
b) to form a basis for appraisal of an ehealth trial (in terms of validity)

CONSORT-EHEALTH items/subitems are MANDATORY reporting items for studies published in the Journal of Medical Internet Research and other journals / scientific societies endorsing the checklist.

Items numbered 1., 2., 3., 4a., 4b etc are original CONSORT or CONSORT-NPT (non-pharmacologic treatment) items.
Items with Roman numerals (i., ii, iii, iv etc.) are CONSORT-EHEALTH extensions/clarifications.

As the CONSORT-EHEALTH checklist is still considered in a formative stage, we would ask that you also RATE ON A SCALE OF 1-5 how important/useful you feel each item is FOR THE PURPOSE OF THE CHECKLIST and reporting guideline (optional).

Mandatory reporting items are marked with a red *.
In the textboxes, either copy & paste the relevant sections from your manuscript into this form - please include any quotes from your manuscript in QUOTATION MARKS,
or answer directly by providing additional information not in the manuscript, or elaborating on why the item was not relevant for this study.

YOUR ANSWERS WILL BE PUBLISHED AS A SUPPLEMENTARY FILE TO YOUR PUBLICATION IN JMIR AND ARE CONSIDERED PART OF YOUR PUBLICATION (IF ACCEPTED).
Please fill in these questions diligently. Information will not be copyedited, so please use proper spelling and grammar, use correct capitalization, and avoid abbreviations.

DO NOT FORGET TO SAVE AS PDF _AND_ CLICK THE SUBMIT BUTTON SO YOUR ANSWERS ARE IN OUR DATABASE !!!

Citation Suggestion (if you append the pdf as Appendix we suggest to cite this paper in the caption):
Eysenbach G, CONSORT-EHEALTH Group
CONSORT-EHEALTH: Improving and Standardizing Evaluation Reports of Web-based and Mobile Health Interventions
J Med Internet Res 2011;13(4):e126
URL: http://www.jmir.org/2011/4/e126/
doi: 10.2196/jmir.1923
PMID: 22209829

Sign in to Google to save your progress. Learn more

* Indicates required question

Your name *

First Last

Caitlin Edwards

Primary Affiliation (short), City, Country *

University of Toronto, Toronto, Canada

Colorado State Univerity, Fort Collins, Coloradc

Your e-mail address *

abc@gmail.com

caitlin.edwards@colostate.edu

Title of your manuscript *

Provide the (draft) title of your manuscript.

A Web-Based Multisite, Pilot and Feasibility Randomized Controlled Trial to Improve Cardiometabolic Health in Adolescent Girls with Elevated Anxiety

Name of your App/Software/Intervention *

If there is a short and a long/alternate name, write the short name first and add the long name in brackets.

Cognitive Behavioral Therapy (CBT), Interpersc

Evaluated Version (if any)

e.g. "V1", "Release 2017-03-01", "Version 2.0.27913"

Your answer

Language(s) *

What language is the intervention/app in? If multiple languages are available, separate by comma (e.g. "English, French")

English

URL of your Intervention Website or App

e.g. a direct link to the mobile app on app in appstore (itunes, Google Play), or URL of the website. If the intervention is a DVD or hardware, you can also link to an Amazon page.

Your answer

URL of an image/screenshot (optional)

Your answer

Accessibility *

Can an enduser access the intervention presently?

- ○ access is free and open
- ○ access only for special usergroups, not open
- ○ access is open to everyone, but requires payment/subscription/in-app purchases
- ◉ app/intervention no longer accessible
- ○ Other:

Primary Medical Indication/Disease/Condition *

e.g. "Stress", "Diabetes", or define the target group in brackets after the condition, e.g. "Autism (Parents of children with)", "Alzheimers (Informal Caregivers of)"

Elevated anxiety (Above Average Weight)

Primary Outcomes measured in trial *

comma-separated list of primary outcomes reported in the trial

Feasibility, Acceptability

Secondary/other outcomes

Are there any other outcomes the intervention is expected to affect?

Describe changes, in anxiety, eating behavior, and cardiometabolic health indices

Recommended "Dose" *

What do the instructions for users say on how often the app should be used?

- ○ Approximately Daily
- ○ Approximately Weekly
- ○ Approximately Monthly
- ○ Approximately Yearly
- ○ "as needed"
- ◉ Other: 12 week virtual group therapy

Approx. Percentage of Users (starters) still using the app as recommended after 3 months *

- ○ unknown / not evaluated
- ○ 0-10%
- ○ 11-20%
- ○ 21-30%
- ○ 31-40%
- ○ 41-50%
- ○ 51-60%
- ○ 61-70%
- ○ 71%-80%
- ○ 81-90%
- ○ 91-100%
- ◉ Other: Not applicable

Overall, was the app/intervention effective? *

- ( ) yes: all primary outcomes were significantly better in intervention group vs control
- ( ) partly: SOME primary outcomes were significantly better in intervention group vs control
- ( ) no statistically significant difference between control and intervention
- ( ) potentially harmful: control was significantly better than intervention in one or more outcomes
- ( ) inconclusive: more research is needed
- (x) Other: Seven out of nine feasibility and acceptability makers were met; no co

Article Preparation Status/Stage *

At which stage in your article preparation are you currently (at the time you fill in this form)

- ( ) not submitted yet - in early draft status
- ( ) not submitted yet - in late draft status, just before submission
- ( ) submitted to a journal but not reviewed yet
- (x) submitted to a journal and after receiving initial reviewer comments
- ( ) submitted to a journal and accepted, but not published yet
- ( ) published
- ( ) Other:

Journal *

If you already know where you will submit this paper (or if it is already submitted), please provide the journal name (if it is not JMIR, provide the journal name under "other")

- ○ not submitted yet / unclear where I will submit this
- ○ Journal of Medical Internet Research (JMIR)
- ○ JMIR mHealth and UHealth
- ○ JMIR Serious Games
- ○ JMIR Mental Health
- ○ JMIR Public Health
- ○ JMIR Formative Research
- ◉ Other JMIR sister journal
- ○ Other:

Is this a full powered effectiveness trial or a pilot/feasibility trial? *

- ◉ Pilot/feasibility
- ○ Fully powered

Manuscript tracking number *

If this is a JMIR submission, please provide the manuscript tracking number under "other" (The ms tracking number can be found in the submission acknowledgement email, or when you login as author in JMIR. If the paper is already published in JMIR, then the ms tracking number is the four-digit number at the end of the DOI, to be found at the bottom of each published article in JMIR)

- ○ no ms number (yet) / not (yet) submitted to / published in JMIR
- ◉ Other: ms#82781

TITLE AND ABSTRACT

1a) TITLE: Identification as a randomized trial in the title

1a) Does your paper address CONSORT item 1a? *

I.e does the title contain the phrase "Randomized Controlled Trial"? (if not, explain the reason under "other")

- ◉ yes
- ○ Other:

1a-i) Identify the mode of delivery in the title

Identify the mode of delivery. Preferably use “web-based” and/or “mobile” and/or “electronic game” in the title. Avoid ambiguous terms like “online”, “virtual”, “interactive”. Use “Internet-based” only if Intervention includes non-web-based Internet components (e.g. email), use “computer-based” or “electronic” only if offline products are used. Use “virtual” only in the context of “virtual reality” (3-D worlds). Use “online” only in the context of “online support groups”. Complement or substitute product names with broader terms for the class of products (such as “mobile” or “smart phone” instead of “iphone”), especially if the application runs on different platforms.

	1	2	3	4	5	
subitem not at all important	○	○	○	◉	○	essential

Clear selection

Does your paper address subitem 1a-i? *

Copy and paste relevant sections from manuscript title (include quotes in quotation marks "like this" to indicate direct quotes from your manuscript), or elaborate on this item by providing additional information not in the ms, or briefly explain why the item is not applicable/relevant for your study

"A Web-Based Multisite, Pilot and Feasibility Randomized Controlled Trial to Improve Cardiometabolic Health in Adolescent Girls with Elevated Anxiety"

1a-ii) Non-web-based components or important co-interventions in title

Mention non-web-based components or important co-interventions in title, if any (e.g., “with telephone support”).

	1	2	3	4	5	
subitem not at all important	◉	○	○	○	○	essential

Clear selection

Does your paper address subitem 1a-ii?

Copy and paste relevant sections from manuscript title (include quotes in quotation marks "like this" to indicate direct quotes from your manuscript), or elaborate on this item by providing additional information not in the ms, or briefly explain why the item is not applicable/relevant for your study

Not applicable, both active conditions were web-based

1a-iii) Primary condition or target group in the title

Mention primary condition or target group in the title, if any (e.g., “for children with Type I Diabetes”) Example: A Web-based and Mobile Intervention with Telephone Support for Children with Type I Diabetes: Randomized Controlled Trial

	1	2	3	4	5	
subitem not at all important	○	○	○	○	◉	essential

Clear selection

Does your paper address subitem 1a-iii? *

Copy and paste relevant sections from manuscript title (include quotes in quotation marks "like this" to indicate direct quotes from your manuscript), or elaborate on this item by providing additional information not in the ms, or briefly explain why the item is not applicable/relevant for your study

"Adolescent Girls with Elevated Anxiety"

1b) ABSTRACT: Structured summary of trial design, methods, results, and conclusions

NPT extension: Description of experimental treatment, comparator, care providers, centers, and blinding status.

1b-i) Key features/functionalities/components of the intervention and comparator in the METHODS section of the ABSTRACT

Mention key features/functionalities/components of the intervention and comparator in the abstract. If possible, also mention theories and principles used for designing the site. Keep in mind the needs of systematic reviewers and indexers by including important synonyms. (Note: Only report in the abstract what the main paper is reporting. If this information is missing from the main body of text, consider adding it)

	1	2	3	4	5	
subitem not at all important	○	○	○	◉	○	essential

Clear selection

Does your paper address subitem 1b-i? *

Copy and paste relevant sections from the manuscript abstract (include quotes in quotation marks "like this" to indicate direct quotes from your manuscript), or elaborate on this item by providing additional information not in the ms, or briefly explain why the item is not applicable/relevant for your study

"Background: Anxiety is common in adolescents with elevated body mass index (BMI), affecting 33-43% of adolescents with overweight/obesity (BMI≥85th percentile for age/sex). Anxiety has been associated with poorer cardiometabolic health, and disinhibited eating in response to anxiety may be a key explanatory mechanism. Therefore, reducing anxiety in adolescents may improve disinhibited eating patterns and prevent worsening cardiometabolic health.

Objective: The primary aims of this pilot study were to assess the feasibility and acceptability of two virtual group psychological interventions, cognitive behavioral therapy (CBT) and interpersonal psychotherapy (IPT), in adolescent girls with elevated anxiety and above-average weight. Secondary/exploratory aims were to describe changes, in anxiety, eating behavior, and cardiometabolic health indices."

1b-ii) Level of human involvement in the METHODS section of the ABSTRACT

Clarify the level of human involvement in the abstract, e.g., use phrases like “fully automated” vs. “therapist/nurse/care provider/physician-assisted” (mention number and expertise of providers involved, if any). (Note: Only report in the abstract what the main paper is reporting. If this information is missing from the main body of text, consider adding it)

	1	2	3	4	5	
subitem not at all important	○	○	●	○	○	essential

Clear selection

Does your paper address subitem 1b-ii?

Copy and paste relevant sections from the manuscript abstract (include quotes in quotation marks "like this" to indicate direct quotes from your manuscript), or elaborate on this item by providing additional information not in the ms, or briefly explain why the item is not applicable/relevant for your study

"Girls were randomly assigned to one of two web-based arms: 12-week, psychologist led, group cognitive-behavioral therapy (CBT) or interpersonal psychotherapy (IPT). In both arms, groups of 5-7 adolescents met for 90 minutes on HIPAA-compliant Zoom, once a week."

1b-iii) Open vs. closed, web-based (self-assessment) vs. face-to-face assessments in the METHODS section of the ABSTRACT

Mention how participants were recruited (online vs. offline), e.g., from an open access website or from a clinic or a closed online user group (closed usergroup trial), and clarify if this was a purely web-based trial, or there were face-to-face components (as part of the intervention or for assessment). Clearly say if outcomes were self-assessed through questionnaires (as common in web-based trials). Note: In traditional offline trials, an open trial (open-label trial) is a type of clinical trial in which both the researchers and participants know which treatment is being administered. To avoid confusion, use “blinded” or “unblinded” to indicated the level of blinding instead of “open”, as “open” in web-based trials usually refers to “open access” (i.e. participants can self-enrol). (Note: Only report in the abstract what the main paper is reporting. If this information is missing from the main body of text, consider adding it)

	1	2	3	4	5	
subitem not at all important	◉	○	○	○	○	essential

Clear selection

Does your paper address subitem 1b-iii?

Copy and paste relevant sections from the manuscript abstract (include quotes in quotation marks "like this" to indicate direct quotes from your manuscript), or elaborate on this item by providing additional information not in the ms, or briefly explain why the item is not applicable/relevant for your study

No, due to limited space and editor and reviewer feedback regarding what should be included in the abstract, recruitment methods are not included.

1b-iv) RESULTS section in abstract must contain use data

Report number of participants enrolled/assessed in each group, the use/uptake of the intervention (e.g., attrition/adherence metrics, use over time, number of logins etc.), in addition to primary/secondary outcomes. (Note: Only report in the abstract what the main paper is reporting. If this information is missing from the main body of text, consider adding it)

	1	2	3	4	5	
subitem not at all important	○	○	○	○	◉	essential

Clear selection

Does your paper address subitem 1b-iv?

Copy and paste relevant sections from the manuscript abstract (include quotes in quotation marks "like this" to indicate direct quotes from your manuscript), or elaborate on this item by providing additional information not in the ms, or briefly explain why the item is not applicable/relevant for your study

"Of eligible adolescents, 79% enrolled. Enrollment of total sample (N = 40) was completed in 7 months. Retention was 85% in IPT and 90% in CBT at 12 week follow up. Protocol adherence exceeded 96% for all intervals Missing data varied, with higher rates for phlebotomy (up to 30%). Acceptability ratings were above average for likability (M±SD 3.03±1.14) and credibility (3.26±.75; 0-4 scale with 4=Highest) across arms. Most adolescents received □ 80% intervention dosage (CBT: 95%, IPT: 90%,). Expert fidelity ratings of CBT were 94±9% and IPT were 78±11%. Both groups demonstrated improvements from baseline to one-year in social anxiety (effect sizes for CBT and IPT were .54 and .72, respectively), disinhibited eating (effect sizes from CBT and IPT were .70 and .46, respectively), and BMI percentile (effect sizes for CBT and IPT were .63 and .82, respectively)."

1b-v) CONCLUSIONS/DISCUSSION in abstract for negative trials

Conclusions/Discussions in abstract for negative trials: Discuss the primary outcome - if the trial is negative (primary outcome not changed), and the intervention was not used, discuss whether negative results are attributable to lack of uptake and discuss reasons. (Note: Only report in the abstract what the main paper is reporting. If this information is missing from the main body of text, consider adding it)

	1	2	3	4	5	
subitem not at all important	○	○	○	◉	○	essential

Clear selection

Does your paper address subitem 1b-v?

Copy and paste relevant sections from the manuscript abstract (include quotes in quotation marks "like this" to indicate direct quotes from your manuscript), or elaborate on this item by providing additional information not in the ms, or briefly explain why the item is not applicable/relevant for your study

"This pilot and feasibility trial provided support for feasibility of recruitment and retention, acceptability of web-based group CBT and IPT, and also yielded areas for optimizing protocol feasibility and intervention fidelity. These preliminary results inform the planning of larger-scale efficacy trials with powered samples to assess effects on BMI and cardiometabolic health indicators among adolescents with elevated anxiety and above-average weight."

INTRODUCTION

2a) In INTRODUCTION: Scientific background and explanation of rationale

2a-i) Problem and the type of system/solution

Describe the problem and the type of system/solution that is object of the study: intended as stand-alone intervention vs. incorporated in broader health care program? Intended for a particular patient population? Goals of the intervention, e.g., being more cost-effective to other interventions, replace or complement other solutions? (Note: Details about the intervention are provided in “Methods” under 5)

	1	2	3	4	5	
subitem not at all important	○	○	○	○	◉	essential

Clear selection

Does your paper address subitem 2a-i? *

Copy and paste relevant sections from the manuscript (include quotes in quotation marks "like this" to indicate direct quotes from your manuscript), or elaborate on this item by providing additional information not in the ms, or briefly explain why the item is not applicable/relevant for your study

"Elevated body mass index (BMI; kg/m²) in adolescents is a serious public health concern. In the United States, about one third of adolescents have overweight/obesity [1] and, globally, adolescent obesity has increased by 1.5% annually over the past decade [2]. Overweight/obesity contributes to earlier manifestation and rising prevalence of cardiometabolic diseases such as type 2 diabetes and cardiovascular diseases [3]. Adolescents with overweight/obesity are more likely to develop insulin resistance, hypertension, dyslipidemia, and prediabetes (Valerio et al., 2024). Such cardiometabolic health concerns disproportionately impact individuals from historically disadvantaged identities [4, 5] and rates are generally higher among girls compared to boys [5]. Moreover, Latinx and Black individuals also experience higher rates of cardiometabolic and cardiovascular health problems across the lifespan, often beginning in adolescence [6], and resulting in a significant disease burden [2, 6].
Adolescence is a sensitive period for the course of excess weight gain and cardiometabolic health [7], as there are significant changes in nutrition, sleep, and physical activity throughout adolescence [8]. Adolescence is also marked by physical changes in body composition that accompany puberty as well as psychosocial changes, including increases in perceived stress [9] and negative affect such as anxiety and depression [10]. Rates of anxiety and depression are increasing among adolescents, with up to 60% of adolescents reporting symptoms of depression and 30% reporting anxiety [11]. Importantly, adolescents with elevated BMI are at higher risk for anxiety disorders, with 33 to 43% of adolescents with overweight/obesity reporting elevated levels of anxiety [12-15]. Anxiety symptoms, especially those associated with social anxiety, commonly manifest during adolescence and are more prevalent in girls [16]. The dynamic physical and psychosocial transitions that characterize adolescence present both a period of heightened risk and an opportunity for impactful intervention to prevent excess weight gain and worsening cardiometabolic health by addressing anxiety."

2a-ii) Scientific background, rationale: What is known about the (type of) system

Scientific background, rationale: What is known about the (type of) system that is the object of the study (be sure to discuss the use of similar systems for other conditions/diagnoses, if appropiate), motivation for the study, i.e. what are the reasons for and what is the context for this specific study, from which stakeholder viewpoint is the study performed, potential impact of findings [2]. Briefly justify the choice of the comparator.

	1	2	3	4	5	
subitem not at all important	○	○	○	○	◉	essential

Clear selection

Does your paper address subitem 2a-ii? *

Copy and paste relevant sections from the manuscript (include quotes in quotation marks "like this" to indicate direct quotes from your manuscript), or elaborate on this item by providing additional information not in the ms, or briefly explain why the item is not applicable/relevant for your study

"The typical behavioral approach to cardiometabolic disease prevention is characterized by one-size-fits-all intensive lifestyle treatment focused on decreasing caloric intake and increasing physical activity. However, such approaches have faced challenges with attrition and sustained effectiveness in adolescents, necessitating more targeted and preventative approaches [17]. As an alternative, addressing psychosocial factors underlying eating and other health behaviors important for weight gain and cardiometabolic health may be particularly suitable for adolescents with above-average BMI.
Anxiety is posited to affect cardiometabolic health through anxiety-induced behaviors. Anxiety is associated with disinhibited eating (i.e., the propensity to overeat in response to stimuli like appetizing foods or negative emotions [18-20]. Indeed, emotional dysregulation, such as anxiety, impacts eating decisions via reward circuitry and interoception; palatable foods become more salient during moments of negative affect [21]. Higher anxiety levels are also associated with more frequent disinhibited eating [22,23], especially loss-of-control eating [24,26], a disordered eating behavior characterized by a perceived inability to control eating regardless of the amount consumed [26]. Furthermore, disinhibited eating patterns, including loss-of-control and eating in response to negative affect, are associated with greater weight gain, adiposity, and higher insulin resistance in youth [25-27]. Thus, anxiety

during adolescence can trigger disinhibited eating leading to excess weight, adiposity, and cardiometabolic health problems.

Frequent anxiety activates also stress-response biobehavioral systems, including the hypothalamic-pituitary adrenal (HPA) axis [28], sympathetic–adrenal–medullary (SAM) system [29], and increased inflammatory response [30]. Repeated and sustained experiences of anxiety contribute to persistent HPA activation, thereby increasing cortisol [31], which leads to visceral fat accumulation, increased gluconeogenesis, endothelial dysfunction, and dyslipidemia [31]. The ongoing activation of the SAM system is associated with increased rates of hypertension, resting heart rate, and decreased heart rate variability [32, 33]. Furthermore, chronic anxiety results in upregulated proinflammatory cytokines [34], thereby lowering insulin resistance, increasing atherogenesis, and impairing glucose control [34]

Treatment for anxiety has been shown to normalize stress physiology [35], improve health behaviors [36], and decrease allostatic load [31]. Furthermore, psychological interventions to address both mental and physical health problems demonstrate greater acceptability than interventions that address mental or physical health concerns [37]. Therefore, anxiety represents a distinct treatment target for preventing further excess weight gain and cardiometabolic problems among adolescents with elevated anxiety and overweight/obesity, particularly by improving disinhibited eating.

Cognitive-behavioral therapy (CBT), the standard-of-care for adolescent anxiety [38] has also been shown to reduce disinhibited eating patterns in adolescents [39] CBT is based on the premise that anxiety is sustained or exacerbated by the interplay of negative thoughts and behavioral avoidance. From this perspective, maladaptive responses to negative affect (e.g., disinhibited eating) are learned behaviors that can be replaced with adaptive cognitive and behavioral coping strategies. CBT involves learning to identify and restructure negative thoughts and attitudes and using behavioral strategies focused on exposure to anxiety-inducing stimuli and rewards for non-avoidant coping [40]. Although CBT is a standard treatment for adolescent anxiety [41], its application to improving weight and cardiometabolic health outcomes in adolescents with elevated anxiety and above-average weight has not yet been tested.

Interpersonal psychotherapy (IPT) offers an alternative to CBT. IPT has been shown to reduce excess weight and adiposity gain over three years compared to didactic controls among adolescent girls with elevated anxiety and above-average BMI [42]. IPT is grounded in interpersonal theory, which holds that social relationships drive negative affect and stress-related behavior. IPT targets four interpersonal problem areas, including grief, role transitions, role disputes, and interpersonal difficulties, that sustain or worsen negative affect [42]. Through facilitated and targeted communication exercises, role plays, and psychoeducation, IPT aims to improve relationships by reducing conflict, enhancing communication, and increasing meaningful support, thereby reducing negative affect and stress-related behaviors [43]. Studies in adolescents with disinhibited eating suggest IPT's promise for reducing loss-of-control eating [44, 45], although IPT has yet to be tested with adolescents with anxiety symptoms specifically.

Extant research has compared the efficacy of CBT and IPT when treating adult

Extant research has compared the efficacy of CBT and IPT when treating adult depression in both randomized clinical trials [46] and in community settings [47], as well as when delivered via telehealth [47]. Generally, CBT and IPT have been found to be equally effective at treating both adult and adolescent anxiety and depression [46, 47]. Indeed, CBT and IPT are considered evidence-based treatments for adolescent mood disorders [489]. Moreover, IPT has been studied in adolescents with disinhibited eating with the aim to prevent excess weight [44] and CBT demonstrates clinically significant improvement for adolescents with eating disorders [49].
However, the intrapersonal emphasis of CBT and the interpersonal focus of IPT have not been specifically compared in adolescents with anxiety and excess weight. Both therapies could be expected to decrease anxiety symptoms and, in turn, reduce excess weight gain and associated cardiometabolic health problems by ameliorating disinhibited eating. Yet, to date, no research has been conducted comparing the efficacy of CBT versus IPT for weight and cardiometabolic health, nor are there data elucidating the potential differing mechanisms of change underlying CBT versus IPT."

2b) In INTRODUCTION: Specific objectives or hypotheses

Does your paper address CONSORT subitem 2b? *

Copy and paste relevant sections from the manuscript (include quotes in quotation marks "like this" to indicate direct quotes from your manuscript), or elaborate on this item by providing additional information not in the ms, or briefly explain why the item is not applicable/relevant for your study

"However, before efficacy or mechanisms can be evaluated, feasibility and acceptability must be established in adolescents with elevated anxiety and overweight/obesity. Multisite studies are ideal for maximizing external validity, timely recruitment of a targeted sample, and capacity for long-term follow-up [50]. Additionally, virtually delivered group psychotherapy is a cost-effective means for increasing access to high-quality mental health care for harder-to-reach communities [51]. Indeed, mental health interventions delivered via telehealth have been found to be effective in managing diverse mental health conditions among adolescents [48, 52]. Furthermore, telementalhealth interventions have been found to reduce mental health stigma [53, 54] and increase access to mental health treatment in historically underserved populations [51, 55, 56],
Therefore, we carried out a two-site, pilot and feasibility randomized controlled trial of group CBT versus group IPT, delivered virtually, for adolescent girls with elevated anxiety and overweight/obesity (IRB #: USUHS.2020-048). In line with pilot study recommendations [57], main outcomes were the feasibility of recruitment, feasibility of research procedures, feasibility of retention, intervention fidelity, and acceptability of interventions."

METHODS

3a) Description of trial design (such as parallel, factorial) including allocation ratio

Does your paper address CONSORT subitem 3a? *

Copy and paste relevant sections from the manuscript (include quotes in quotation marks "like this" to indicate direct quotes from your manuscript), or elaborate on this item by providing additional information not in the ms, or briefly explain why the item is not applicable/relevant for your study

No, as this was a two arm pilot and feasibility trial with two active interventions. However, we do discuss randomization and blinding: "Eligible participants were randomly assigned to CBT or IPT. Participants were randomized using a computerized number generator created by a statistician; only the statistician knew the sequence. Randomization was performed by the statistician only after eligibility was determined. Every consecutive block of 2-7 girls at each location were randomized to CBT or IPT. Randomization continued until a full cohort (4-7 adolescents per group arm) was created. Neither interventionists, by design, nor outcome assessors were blind to assignment, except for research staff performing phlebotomy who were blind."

3b) Important changes to methods after trial commencement (such as eligibility criteria), with reasons

Does your paper address CONSORT subitem 3b? *

Copy and paste relevant sections from the manuscript (include quotes in quotation marks "like this" to indicate direct quotes from your manuscript), or elaborate on this item by providing additional information not in the ms, or briefly explain why the item is not applicable/relevant for your study

Not applicable; there were no substantial changes to methods after trial was implemented.

3b-i) Bug fixes, Downtimes, Content Changes

Bug fixes, Downtimes, Content Changes: ehealth systems are often dynamic systems. A description of changes to methods therefore also includes important changes made on the intervention or comparator during the trial (e.g., major bug fixes or changes in the functionality or content) (5-iii) and other “unexpected events” that may have influenced study design such as staff changes, system failures/downtimes, etc. [2].

	1	2	3	4	5	
subitem not at all important	◉	○	○	○	○	essential

Clear selection

Does your paper address subitem 3b-i?

Copy and paste relevant sections from the manuscript (include quotes in quotation marks "like this" to indicate direct quotes from your manuscript), or elaborate on this item by providing additional information not in the ms, or briefly explain why the item is not applicable/relevant for your study

Not applicable; interventions took place over HIPAA compliant Zoom and no problems were reported.

4a) Eligibility criteria for participants

Does your paper address CONSORT subitem 4a? *

Copy and paste relevant sections from the manuscript (include quotes in quotation marks "like this" to indicate direct quotes from your manuscript), or elaborate on this item by providing additional information not in the ms, or briefly explain why the item is not applicable/relevant for your study

"Enrollment for this pilot and feasibility randomized controlled trial was carried out at two sites: (1) the Uniformed Services University (USU) in Bethesda, Maryland, and (2) Colorado State University (CSU) in Fort Collins, Colorado. Participants were girls aged 12 to 17 years with elevated anxiety symptoms, as indicated by a total score ≥32 on the State-Trait Anxiety Inventory for Children [STAI-C; 58] and above-average BMI (≥75th percentile for age/sex). Exclusion criteria included any major medical conditions, pregnancy or breastfeeding, regular medication use likely to impact mood or weight (e.g., insulin sensitizers, GLP-1 receptor agonists, antidepressants, stimulants), current involvement in psychotherapy, and a psychiatric disorder or symptoms (e.g., active suicidal ideation, behavior, or self-harm) that, in the opinion of the investigators, would impede competence, compliance, or otherwise hinder completion of the study. Exclusion criteria were chosen to ensure adolescents who needed a higher level of care—beyond what could be provided in the context of a research study—were not included and any medications or interventions that could impact anxiety, eating behaviors, and/or cardiometabolic health were removed to mitigate confounding variables. If participants developed exclusionary criteria after randomization, they were withdrawn if participation was counter-indicated for safety or validity, in the opinion of the investigators."

4a-i) Computer / Internet literacy

Computer / Internet literacy is often an implicit “de facto” eligibility criterion - this should be explicitly clarified.

	1	2	3	4	5	
subitem not at all important	○	◉	○	○	○	essential

Clear selection

Does your paper address subitem 4a-i?

Copy and paste relevant sections from the manuscript (include quotes in quotation marks "like this" to indicate direct quotes from your manuscript), or elaborate on this item by providing additional information not in the ms, or briefly explain why the item is not applicable/relevant for your study

This item is not included in the manuscript; as adolescents could be recruited and seen for screening visits in person with their parents/guardians, they only needed to have access to Zoom for the intervention. Thus, any adolescent who did not have access to the internet was automatically excluded and this was not reported to be a problem by study personnel.

4a-ii) Open vs. closed, web-based vs. face-to-face assessments:

Open vs. closed, web-based vs. face-to-face assessments: Mention how participants were recruited (online vs. offline), e.g., from an open access website or from a clinic, and clarify if this was a purely web-based trial, or there were face-to-face components (as part of the intervention or for assessment), i.e., to what degree got the study team to know the participant. In online-only trials, clarify if participants were quasi-anonymous and whether having multiple identities was possible or whether technical or logistical measures (e.g., cookies, email confirmation, phone calls) were used to detect/prevent these.

	1	2	3	4	5	
subitem not at all important	○	○	○	○	●	essential

Clear selection

Does your paper address subitem 4a-ii? *

Copy and paste relevant sections from the manuscript (include quotes in quotation marks "like this" to indicate direct quotes from your manuscript), or elaborate on this item by providing additional information not in the ms, or briefly explain why the item is not applicable/relevant for your study

"Recruitment took place from February 2021 to August 2021. Letters and flyers were sent to parents of girls aged 12 to 17 years who resided within 60 miles of USU or CSU; letters were also mailed to local pediatricians and family physicians. The study was advertised on school and parent email listservs, in local newspapers, and on social media. The target sample size was 40 participants (20 per site), in line with recommendations for pilot studies in which the primary effect size in future efficacy trials is expected to be small to moderate [59].
Adolescents and their parents initially completed a phone pre-screening to estimate eligibility. Adolescents who were interested and appeared eligible were scheduled for an in-person screening and baseline data collection visit at either USU or CSU. The screening visit included written consent and assent, body measurements and fasting blood draw, brief psychiatric and medical health history with the participant and parent/guardian, interview, and surveys. Medical health history included age of menarche and menstrual cycle status. Adolescents completed surveys via a secure electronic data-capture system (e.g., Research Electronic Data Capture (REDCap), while parents completed questionnaires about parental education (highest education attained) and subjective socioeconomic status using the MacArthur Scale of Subjective Social Status [60]. Questionnaires, such as the family history form, not previously validated with other populations (e.g., the MacArthur Scale of Subjective Social Status [60]), were developed by principal investigators and statisticians and piloted with study staff. Surveys were password protected; passwords were only known by study staff. Prior to completing any questionnaires, both parents and adolescents were informed of the length of the time of the surveys, where data would be stored (e.g., REDCap), the name of the principal investigators, and the purpose of the questionnaires. As all surveys consisted of pre-specified measures, randomization of questions and adaptive questionnaires were not used, and incomplete questionnaires were included in analysis."

4a-iii) Information giving during recruitment

Information given during recruitment. Specify how participants were briefed for recruitment and in the informed consent procedures (e.g., publish the informed consent documentation as appendix, see also item X26), as this information may have an effect on user self-selection, user expectation and may also bias results.

	1	2	3	4	5	
subitem not at all important	○	○	○	●	○	essential

Clear selection

Does your paper address subitem 4a-iii?

Copy and paste relevant sections from the manuscript (include quotes in quotation marks "like this" to indicate direct quotes from your manuscript), or elaborate on this item by providing additional information not in the ms, or briefly explain why the item is not applicable/relevant for your study

"The use of human subjects in this research was reviewed and approved by the Uniformed Services University Investigative Review Board (IRB #: USUHS.2020-048). Adolescents and parents/guardians were provided with informed consent and assent documents by trained study staff in the first screening visit. Informed consent documents included information on the nature and purpose of the study, study procedures including as at home device wear and group interventions, confidentiality, study benefits, compensation, study risks, alternative therapy programs, and contact information if concerns arose. Adolescents were compensated for participating in various aspects of the study; in total, they could earn up to $917 if they completed all visits, at home data collection, surveys, group sessions, and follow up visits. All data collected were de-identified."

4b) Settings and locations where the data were collected

Does your paper address CONSORT subitem 4b? *

Copy and paste relevant sections from the manuscript (include quotes in quotation marks "like this" to indicate direct quotes from your manuscript), or elaborate on this item by providing additional information not in the ms, or briefly explain why the item is not applicable/relevant for your study

"Uniformed Services University" "Colorado State University" "HIPAA compliant Zoom"

4b-i) Report if outcomes were (self-)assessed through online questionnaires

Clearly report if outcomes were (self-)assessed through online questionnaires (as common in web-based trials) or otherwise.

	1	2	3	4	5	
subitem not at all important	○	○	○	◉	○	essential

Clear selection

Does your paper address subitem 4b-i? *

Copy and paste relevant sections from the manuscript (include quotes in quotation marks "like this" to indicate direct quotes from your manuscript), or elaborate on this item by providing additional information not in the ms, or briefly explain why the item is not applicable/relevant for your study

"Adolescents completed surveys via a secure electronic data-capture system (e.g., Research Electronic Data Capture (REDCap), while parents completed questionnaires about parental education (highest education attained) and subjective socioeconomic status using the MacArthur Scale of Subjective Social Status [60]."

4b-ii) Report how institutional affiliations are displayed

Report how institutional affiliations are displayed to potential participants [on ehealth media], as affiliations with prestigious hospitals or universities may affect volunteer rates, use, and reactions with regards to an intervention.(Not a required item – describe only if this may bias results)

	1	2	3	4	5	
subitem not at all important	(selected)	○	○	○	○	essential

Clear selection

Does your paper address subitem 4b-ii?

Copy and paste relevant sections from the manuscript (include quotes in quotation marks "like this" to indicate direct quotes from your manuscript), or elaborate on this item by providing additional information not in the ms, or briefly explain why the item is not applicable/relevant for your study

Your answer

5) The interventions for each group with sufficient details to allow replication, including how and when they were actually administered

5-i) Mention names, credential, affiliations of the developers, sponsors, and owners

Mention names, credential, affiliations of the developers, sponsors, and owners [6] (if authors/evaluators are owners or developer of the software, this needs to be declared in a "Conflict of interest" section or mentioned elsewhere in the manuscript).

	1	2	3	4	5	
subitem not at all important	◉	○	○	○	○	essential

Clear selection

Does your paper address subitem 5-i?

Copy and paste relevant sections from the manuscript (include quotes in quotation marks "like this" to indicate direct quotes from your manuscript), or elaborate on this item by providing additional information not in the ms, or briefly explain why the item is not applicable/relevant for your study

Multiple developers of both cognitive behavioral therapy and interpersonal psychotherapy

5-ii) Describe the history/development process

Describe the history/development process of the application and previous formative evaluations (e.g., focus groups, usability testing), as these will have an impact on adoption/use rates and help with interpreting results.

	1	2	3	4	5	
subitem not at all important	◉	○	○	○	○	essential

Clear selection

Does your paper address subitem 5-ii?

Copy and paste relevant sections from the manuscript (include quotes in quotation marks "like this" to indicate direct quotes from your manuscript), or elaborate on this item by providing additional information not in the ms, or briefly explain why the item is not applicable/relevant for your study

Both cognitive behavioral therapy and interpersonal psychotherapy have been in ongoing development for decades

5-iii) Revisions and updating

Revisions and updating. Clearly mention the date and/or version number of the application/intervention (and comparator, if applicable) evaluated, or describe whether the intervention underwent major changes during the evaluation process, or whether the development and/or content was “frozen” during the trial. Describe dynamic components such as news feeds or changing content which may have an impact on the replicability of the intervention (for unexpected events see item 3b).

	1	2	3	4	5	
subitem not at all important	(●)	○	○	○	○	essential

Clear selection

Does your paper address subitem 5-iii?

Copy and paste relevant sections from the manuscript (include quotes in quotation marks "like this" to indicate direct quotes from your manuscript), or elaborate on this item by providing additional information not in the ms, or briefly explain why the item is not applicable/relevant for your study

Not applicable; no version number

5-iv) Quality assurance methods

Provide information on quality assurance methods to ensure accuracy and quality of information provided [1], if applicable.

	1	2	3	4	5	
subitem not at all important	○	○	●	○	○	essential

Clear selection

Does your paper address subitem 5-iv?

Copy and paste relevant sections from the manuscript (include quotes in quotation marks "like this" to indicate direct quotes from your manuscript), or elaborate on this item by providing additional information not in the ms, or briefly explain why the item is not applicable/relevant for your study

"Intervention fidelity was assessed via fidelity rating scales. Fidelity rating scales were adapted from previously developed CBT and IPT program adherence measures provided by the study’s primary investigators by two PhD-level mental/behavioral health clinician-researchers not involved in intervention delivery and/or supervision. Measures were developed to reflect session focused tasks specified in CBT and IPT intervention manuals. The mental/behavioral health clinician-researchers met consistently with experts in CBT and IPT over two months to develop the fidelity measures. Fidelity measures were pilot tested using randomly chosen audio-recorded CBT and IPT sessions. Inter-rater reliability during pilot testing ranged between .95 and 1 (intraclass correlation coefficient, 0.975; 95% CI, 0.95-1.00)."

5-v) Ensure replicability by publishing the source code, and/or providing screenshots/screen-capture video, and/or providing flowcharts of the algorithms used

Ensure replicability by publishing the source code, and/or providing screenshots/screen-capture video, and/or providing flowcharts of the algorithms used. Replicability (i.e., other researchers should in principle be able to replicate the study) is a hallmark of scientific reporting.

	1	2	3	4	5	
subitem not at all important	(●)	( )	( )	( )	( )	essential

Clear selection

Does your paper address subitem 5-v?

Copy and paste relevant sections from the manuscript (include quotes in quotation marks "like this" to indicate direct quotes from your manuscript), or elaborate on this item by providing additional information not in the ms, or briefly explain why the item is not applicable/relevant for your study

Not applicable; no source code

5-vi) Digital preservation

Digital preservation: Provide the URL of the application, but as the intervention is likely to change or disappear over the course of the years; also make sure the intervention is archived (Internet Archive, webcitation.org, and/or publishing the source code or screenshots/videos alongside the article). As pages behind login screens cannot be archived, consider creating demo pages which are accessible without login.

	1	2	3	4	5	
subitem not at all important	(•)	( )	( )	( )	( )	essential

Clear selection

Does your paper address subitem 5-vi?

Copy and paste relevant sections from the manuscript (include quotes in quotation marks "like this" to indicate direct quotes from your manuscript), or elaborate on this item by providing additional information not in the ms, or briefly explain why the item is not applicable/relevant for your study

Not applicable; no URL of application

5-vii) Access

Access: Describe how participants accessed the application, in what setting/context, if they had to pay (or were paid) or not, whether they had to be a member of specific group. If known, describe how participants obtained “access to the platform and Internet” [1]. To ensure access for editors/reviewers/readers, consider to provide a “backdoor” login account or demo mode for reviewers/readers to explore the application (also important for archiving purposes, see vi).

	1	2	3	4	5	
subitem not at all important	○	○	○	○	○	essential

Does your paper address subitem 5-vii? *

Copy and paste relevant sections from the manuscript (include quotes in quotation marks "like this" to indicate direct quotes from your manuscript), or elaborate on this item by providing additional information not in the ms, or briefly explain why the item is not applicable/relevant for your study

Not applicable; study staff provided a HIPAA compliant Zoom link

5-viii) Mode of delivery, features/functionalities/components of the intervention and comparator, and the theoretical framework

Describe mode of delivery, features/functionalities/components of the intervention and comparator, and the theoretical framework [6] used to design them (instructional strategy [1], behaviour change techniques, persuasive features, etc., see e.g., [7, 8] for terminology). This includes an in-depth description of the content (including where it is coming from and who developed it) [1]," whether [and how] it is tailored to individual circumstances and allows users to track their progress and receive feedback" [6]. This also includes a description of communication delivery channels and – if computer-mediated communication is a component – whether communication was synchronous or asynchronous [6]. It also includes information on presentation strategies [1], including page design principles, average amount of text on pages, presence of hyperlinks to other resources, etc. [1].

	1	2	3	4	5	
subitem not at all important	○	○	○	○	◉	essential

Clear selection

Does your paper address subitem 5-viii? *

Copy and paste relevant sections from the manuscript (include quotes in quotation marks "like this" to indicate direct quotes from your manuscript), or elaborate on this item by providing additional information not in the ms, or briefly explain why the item is not applicable/relevant for your study

"Cognitive-behavioral therapy (CBT), the standard-of-care for adolescent anxiety [38] has also been shown to reduce disinhibited eating patterns in adolescents [39] CBT is based on the premise that anxiety is sustained or exacerbated by the interplay of negative thoughts and behavioral avoidance. From this perspective, maladaptive responses to negative affect (e.g., disinhibited eating) are learned behaviors that can be replaced with adaptive cognitive and behavioral coping strategies. CBT involves learning to identify and restructure negative thoughts and attitudes and using behavioral strategies focused on exposure to anxiety-inducing stimuli and rewards for non-avoidant coping [40]. Although CBT is a standard treatment for adolescent anxiety [41], its application to improving weight and cardiometabolic health outcomes in adolescents with elevated anxiety and above-average weight has not yet been tested.

Interpersonal psychotherapy (IPT) offers an alternative to CBT. IPT has been shown to reduce excess weight and adiposity gain over three years compared to didactic controls among adolescent girls with elevated anxiety and above-average BMI [42]. IPT is grounded in interpersonal theory, which holds that social relationships drive negative affect and stress-related behavior. IPT targets four interpersonal problem areas, including grief, role transitions, role disputes, and interpersonal difficulties, that sustain or worsen negative affect [42]. Through facilitated and targeted communication exercises, role plays, and psychoeducation, IPT aims to improve relationships by reducing conflict, enhancing communication, and increasing meaningful support, thereby reducing negative affect and stress-related behaviors [43]. Studies in adolescents with disinhibited eating suggest IPT's promise for reducing loss-of-control eating [44, 45], although IPT has yet to be tested with adolescents with anxiety symptoms specifically.

Extant research has compared the efficacy of CBT and IPT when treating adult depression in both randomized clinical trials [46] and in community settings [47], as well as when delivered via telehealth [47]. Generally, CBT and IPT have been found to be equally effective at treating both adult and adolescent anxiety and depression [46, 47]. Indeed, CBT and IPT are considered evidence-based treatments for adolescent mood disorders [489]. Moreover, IPT has been studied in adolescents with disinhibited eating with the aim to prevent excess weight [44] and CBT demonstrates clinically significant improvement for adolescents with eating disorders [49]."

5-ix) Describe use parameters

Describe use parameters (e.g., intended “doses” and optimal timing for use). Clarify what instructions or recommendations were given to the user, e.g., regarding timing, frequency, heaviness of use, if any, or was the intervention used ad libitum.

	1	2	3	4	5	
subitem not at all important	○	○	○	○	◉	essential

Clear selection

Does your paper address subitem 5-ix?

Copy and paste relevant sections from the manuscript (include quotes in quotation marks "like this" to indicate direct quotes from your manuscript), or elaborate on this item by providing additional information not in the ms, or briefly explain why the item is not applicable/relevant for your study

"Sessions lasted 90 minutes and took place weekly after school hours for 12 weeks."

5-x) Clarify the level of human involvement

Clarify the level of human involvement (care providers or health professionals, also technical assistance) in the e-intervention or as co-intervention (detail number and expertise of professionals involved, if any, as well as “type of assistance offered, the timing and frequency of the support, how it is initiated, and the medium by which the assistance is delivered”. It may be necessary to distinguish between the level of human involvement required for the trial, and the level of human involvement required for a routine application outside of a RCT setting (discuss under item 21 – generalizability).

	1	2	3	4	5	
subitem not at all important	○	○	○	○	◉	essential

Clear selection

Does your paper address subitem 5-x?

Copy and paste relevant sections from the manuscript (include quotes in quotation marks "like this" to indicate direct quotes from your manuscript), or elaborate on this item by providing additional information not in the ms, or briefly explain why the item is not applicable/relevant for your study

"All virtual groups were co-facilitated by a psychologist and a graduate student with clinical training via HIPAA compliant Zoom; both facilitators conducted the CBT and IPT groups. Reminder emails were sent 24 hours prior to each group."

5-xi) Report any prompts/reminders used

Report any prompts/reminders used: Clarify if there were prompts (letters, emails, phone calls, SMS) to use the application, what triggered them, frequency etc. It may be necessary to distinguish between the level of prompts/reminders required for the trial, and the level of prompts/reminders for a routine application outside of a RCT setting (discuss under item 21 – generalizability).

	1	2	3	4	5	
subitem not at all important	○	○	○	◉	○	essential

Clear selection

Does your paper address subitem 5-xi? *

Copy and paste relevant sections from the manuscript (include quotes in quotation marks "like this" to indicate direct quotes from your manuscript), or elaborate on this item by providing additional information not in the ms, or briefly explain why the item is not applicable/relevant for your study

"Reminder emails were sent 24 hours prior to each group."

5-xii) Describe any co-interventions (incl. training/support)

Describe any co-interventions (incl. training/support): Clearly state any interventions that are provided in addition to the targeted eHealth intervention, as ehealth intervention may not be designed as stand-alone intervention. This includes training sessions and support [1]. It may be necessary to distinguish between the level of training required for the trial, and the level of training for a routine application outside of a RCT setting (discuss under item 21 – generalizability.

	1	2	3	4	5	
subitem not at all important	(•)	( )	( )	( )	( )	essential

Clear selection

Does your paper address subitem 5-xii? *

Copy and paste relevant sections from the manuscript (include quotes in quotation marks "like this" to indicate direct quotes from your manuscript), or elaborate on this item by providing additional information not in the ms, or briefly explain why the item is not applicable/relevant for your study

Not applicable; no additional training or support

6a) Completely defined pre-specified primary and secondary outcome measures, including how and when they were assessed

Does your paper address CONSORT subitem 6a? *

Copy and paste relevant sections from the manuscript (include quotes in quotation marks "like this" to indicate direct quotes from your manuscript), or elaborate on this item by providing additional information not in the ms, or briefly explain why the item is not applicable/relevant for your study

"Study flow, including time to enroll the total target sample, was tracked following CONSORT guidelines for pilot studies [61]. Recruitment feasibility was operationalized as enrollment of the target sample size (N = 40) within 12 months. The percentage of eligible youth who enrolled was also calculated. Retention was tracked at each follow-up interval. Benchmarks for retention feasibility were defined as ≥80% retention at post-treatment/12-week follow-up and ≥70% retention at one-year follow-up. Feasibility of the study protocol was assessed by research staff completion of study flow checklists for standardized operating procedures at each interval. A threshold of ≥90% adherence to standardized procedures was expected, with ≤5% missing data for surveys, interviews, body measurements, blood pressure, and blood collection among completers at each interval.
Primary Aims
Primary study aims were recruitment and retention feasibility, study protocol/data collection feasibility, intervention fidelity, and program acceptability. Recruitment and retention feasibility were assessed through timely recruitment, attendance, and retention at 12 week and one year follow up. Intervention fidelity was assessed via fidelity rating scales. Fidelity rating scales were adapted from previously developed CBT and IPT program adherence measures provided by the study's primary investigators by two PhD-level mental/behavioral health clinician-researchers not involved in intervention delivery and/or supervision. Measures were developed to reflect session focused tasks specified in CBT and IPT intervention manuals. The mental/behavioral health clinician-researchers met consistently with experts in CBT and IPT over two months to develop the fidelity measures. Fidelity measures were pilot tested using randomly chosen audio-recorded CBT and IPT sessions. Inter-rater reliability during pilot testing ranged between .95 and 1 (intraclass correlation coefficient, 0.975; 95% CI, 0.95-1.00).
Intervention fidelity was assessed through review of 20% of randomly selected audio-recorded CBT and IPT sessions by the two PhD-level mental/behavioral health clinician-researchers. In line with existing fidelity research [62], a benchmark of ≥80% adherence was set. CBT checklist examples included: "Present a scenario where teens cope successfully with a problem and provide self-ratings." Items were rated 1 = Yes, 0.5 = Partially, or 0 = No, with percentage fidelity calculated as the average adherence across all items and sessions per arm. The IPT fidelity checklist included items such as: "Have ≥1 group member discuss an interpersonal situation from the previous week and have the group ask questions; facilitate communication analysis if indicated." Interrater reliability for six co-rated sessions was excellent (intraclass correlation coefficient, 0.96; 95% CI, 0.92-0.98).
To assess intervention acceptability, at 12-week follow-up, adolescents completed an adapted Treatment Acceptability Questionnaire [TAQ; 63] which measures likeability and credibility of CBT and IPT on a Likert scale (0 = Not at all to 4 = Definitely). Acceptability was defined as an average score ≥3 on likeability/credibility ratings within each arm. Acceptability was also characterized by attendance; ≥80% attendance at ≥10 of 12 group sessions was anticipated in both arms.

Secondary Aims
Secondary aims were to describe within-arm changes in anxiety symptoms, disinhibited

Secondary aims were to describe within-arm changes in anxiety symptoms, disinhibited eating (emotional eating and loss-of-control eating), BMI indices and adiposity, and cardiometabolic health indicators including blood pressure, cholesterol, triglycerides, glucose, and HbA1c. Anxiety and eating behaviors were collected through online survey self-assessments, with the exception of the Eating Disorder Examination–Overeating Section [EDE-O; 64], which was collected via interview by trained research staff. The STAI-C [65] assessed eligibility (total score ≥32; range 20-60) and anxiety symptom severity. The STAI-C [65] has been validated for screening anxiety in youth with chronic health conditions with good psychometric properties [66]. Anxiety was measured at baseline and one-year follow-up. Given the relevance of social anxiety in adolescents with elevated BMI, participants also completed the Social Phobia and Anxiety Inventory for Children [SPAI-C; 67] a 26-item self-report assessing somatic, cognitive, and behavioral aspects of social anxiety. Higher total scores (range 0-52) indicate more symptoms. The SPAI-C [67] is validated in children aged 8-17 years with good psychometric properties [67] and was administered at baseline, 12-week, and one-year follow-ups.
The Emotional Eating Scale Adapted for Children and Adolescents [EES-C; 68] is a 25-item self-report assessing urge to eat in response to negative affect. Total scores (range 25-100) were averaged. The EES-C [68] demonstrates good internal consistency and validity [69].
Loss-of-control eating was assessed via the Eating Disorder Examination–Overeating Section [EDE-O; 64] administered by trained staff. Frequency of loss-of-control episodes in the past month was recorded as total episodes characterized by subjective inability to control eating any amount of food (subjective and/or objective binge-eating episodes).
Height was measured in triplicate with a stadiometer (Charder Electronic COLTD; Model HM 200P) and averaged. Fasting weight was measured on a calibrated digital scale connected to a Bod Pod [70]. Height and weight were used to calculate BMI indices [71]. Body fat percentage was measured via air displacement plethysmography using Bod Pod in a fasted state with minimal clothing and a swim cap [70, 72].
Fasting blood samples were collected to measure glucose, cholesterol, triglycerides, and glycated hemoglobin (HbA1c). Resting blood pressure was measured in triplicate with an automatic monitor (LifeSource; Model UA-789AC) and averaged. Blood samples were processed at the University of Colorado Anschutz Medical Campus Core Lab using standard procedures.
CONSORT study flow information and descriptive summary statistics were used to assess recruitment and retention feasibility, study protocol/data collection feasibility, intervention fidelity, and program acceptability. Group medians, interquartile ranges, and 95% confidence intervals (CIs) were reported at each measurement interval, including baseline, 12-week follow-up, and one-year follow-up, to illustrate patterns within arms over time. These descriptive data include all participants who completed each specific time point, resulting in the possibility of different samples sizes at each timepoint. Multiple imputation was not used given that it relies on patterns in the data and therefore may be noisy or unreliable given the small sample size per group [74]. To describe changes in clinical health-related outcomes, change scores from baseline to 12-week follow-up and baseline to one-year follow-up were examined in each arm (CBT and IPT) separately, and this analysis included

participants who completed both time points needed for calculation of the change score (i.e., baseline and 12 week; baseline and 1 year). Given the pilot nature of the study, in which we were focused on the estimation of a signal, a one-sample t-test was used to compare to a non-zero benchmark. One-sample t-tests were used for normally distributed outcomes, and Wilcoxon signed rank tests were used when outcomes deviated from normality. Cohen's d was calculated to describe effect sizes for normally distributed outcomes, while r was used for non-normal outcomes. For acceptability ratings, Mann-Whitney tests were used to test for potential group differences across domains of acceptability."

6a-i) Online questionnaires: describe if they were validated for online use and apply CHERRIES items to describe how the questionnaires were designed/deployed

If outcomes were obtained through online questionnaires, describe if they were validated for online use and apply CHERRIES items to describe how the questionnaires were designed/deployed [9].

	1	2	3	4	5	
subitem not at all important	○	○	◉	○	○	essential

Clear selection

Does your paper address subitem 6a-i?

Copy and paste relevant sections from manuscript text

"Adolescents completed surveys via a secure electronic data-capture system (e.g., Research Electronic Data Capture (REDCap), while parents completed questionnaires about parental education (highest education attained) and subjective socioeconomic status using the MacArthur Scale of Subjective Social Status [60]. Questionnaires, such as the family history form, not previously validated with other populations (e.g., the MacArthur Scale of Subjective Social Status [60]), were developed by principal investigators and statisticians and piloted with study staff. Surveys were password protected; passwords were only known by study staff. Prior to completing any questionnaires, both parents and adolescents were informed of the length of the time of the surveys, where data would be stored (e.g., REDCap), the name of the principal investigators, and the purpose of the questionnaires. As all surveys consisted of pre-specified measures, randomization of questions and adaptive questionnaires were not used, and incomplete questionnaires were included in analysis."

6a-ii) Describe whether and how “use” (including intensity of use/dosage) was defined/measured/monitored

Describe whether and how “use” (including intensity of use/dosage) was defined/measured/monitored (logins, logfile analysis, etc.). Use/adoption metrics are important process outcomes that should be reported in any ehealth trial.

	1	2	3	4	5	
subitem not at all important	○	○	○	○	●	essential

Clear selection

Does your paper address subitem 6a-ii?

Copy and paste relevant sections from manuscript text

"Acceptability was also characterized by attendance; ≥80% attendance at ≥10 of 12 group sessions was anticipated in both arms."

6a-iii) Describe whether, how, and when qualitative feedback from participants was obtained

Describe whether, how, and when qualitative feedback from participants was obtained (e.g., through emails, feedback forms, interviews, focus groups).

	1	2	3	4	5	
subitem not at all important	(●)	( )	( )	( )	( )	essential

Clear selection

Does your paper address subitem 6a-iii?

Copy and paste relevant sections from manuscript text

Not applicable; qualitative feedback was not obtained for this RCT

6b) Any changes to trial outcomes after the trial commenced, with reasons

Does your paper address CONSORT subitem 6b? *

Copy and paste relevant sections from the manuscript (include quotes in quotation marks "like this" to indicate direct quotes from your manuscript), or elaborate on this item by providing additional information not in the ms, or briefly explain why the item is not applicable/relevant for your study

Not applicable; no changes to trial outcomes occurred after trial commenced

7a) How sample size was determined

NPT: When applicable, details of whether and how the clustering by care provides or centers was addressed

7a-i) Describe whether and how expected attrition was taken into account when calculating the sample size

Describe whether and how expected attrition was taken into account when calculating the sample size.

	1	2	3	4	5	
subitem not at all important	○	○	○	○	◉	essential

Clear selection

Does your paper address subitem 7a-i?

Copy and paste relevant sections from manuscript title (include quotes in quotation marks "like this" to indicate direct quotes from your manuscript), or elaborate on this item by providing additional information not in the ms, or briefly explain why the item is not applicable/relevant for your study

"The target sample size was 40 participants (20 per site), in line with recommendations for pilot studies in which the primary effect size in future efficacy trials is expected to be small to moderate [59]."

7b) When applicable, explanation of any interim analyses and stopping guidelines

Does your paper address CONSORT subitem 7b? *

Copy and paste relevant sections from the manuscript (include quotes in quotation marks "like this" to indicate direct quotes from your manuscript), or elaborate on this item by providing additional information not in the ms, or briefly explain why the item is not applicable/relevant for your study

Not applicable; no interim analyses occurred

8a) Method used to generate the random allocation sequence

NPT: When applicable, how care providers were allocated to each trial group

Does your paper address CONSORT subitem 8a? *

Copy and paste relevant sections from the manuscript (include quotes in quotation marks "like this" to indicate direct quotes from your manuscript), or elaborate on this item by providing additional information not in the ms, or briefly explain why the item is not applicable/relevant for your study

"Eligible participants were randomly assigned to CBT or IPT. Participants were randomized using a computerized number generator created by a statistician; only the statistician knew the sequence. Randomization was performed by the statistician only after eligibility was determined. Every consecutive block of 2-7 girls at each location were randomized to CBT or IPT. Randomization continued until a full cohort (4-7 adolescents per group arm) was created. Neither interventionists, by design, nor outcome assessors were blind to assignment, except for research staff performing phlebotomy who were blind."

8b) Type of randomisation; details of any restriction (such as blocking and block size)

Does your paper address CONSORT subitem 8b? *

Copy and paste relevant sections from the manuscript (include quotes in quotation marks "like this" to indicate direct quotes from your manuscript), or elaborate on this item by providing additional information not in the ms, or briefly explain why the item is not applicable/relevant for your study

"Every consecutive block of 2-7 girls at each location were randomized to CBT or IPT."

9) Mechanism used to implement the random allocation sequence (such as sequentially numbered containers), describing any steps taken to conceal the sequence until interventions were assigned

Does your paper address CONSORT subitem 9? *

Copy and paste relevant sections from the manuscript (include quotes in quotation marks "like this" to indicate direct quotes from your manuscript), or elaborate on this item by providing additional information not in the ms, or briefly explain why the item is not applicable/relevant for your study

"Participants were randomized using a computerized number generator created by a statistician; only the statistician knew the sequence."

10) Who generated the random allocation sequence, who enrolled participants, and who assigned participants to interventions

Does your paper address CONSORT subitem 10? *

Copy and paste relevant sections from the manuscript (include quotes in quotation marks "like this" to indicate direct quotes from your manuscript), or elaborate on this item by providing additional information not in the ms, or briefly explain why the item is not applicable/relevant for your study

Study statistician generated the random allocation sequence, study staff enrolled participants as well as assigned them to interventions

11a) If done, who was blinded after assignment to interventions (for example, participants, care providers, those assessing outcomes) and how

NPT: Whether or not administering co-interventions were blinded to group assignment

11a-i) Specify who was blinded, and who wasn't

Specify who was blinded, and who wasn't. Usually, in web-based trials it is not possible to blind the participants [1, 3] (this should be clearly acknowledged), but it may be possible to blind outcome assessors, those doing data analysis or those administering co-interventions (if any).

	1	2	3	4	5	
subitem not at all important	○	○	○	○	○	essential

Does your paper address subitem 11a-i? *

Copy and paste relevant sections from the manuscript (include quotes in quotation marks "like this" to indicate direct quotes from your manuscript), or elaborate on this item by providing additional information not in the ms, or briefly explain why the item is not applicable/relevant for your study

"Neither interventionists, by design, nor outcome assessors were blind to assignment, except for research staff performing phlebotomy who were blind."

11a-ii) Discuss e.g., whether participants knew which intervention was the "intervention of interest" and which one was the "comparator"

Informed consent procedures (4a-ii) can create biases and certain expectations - discuss e.g., whether participants knew which intervention was the "intervention of interest" and which one was the "comparator".

	1	2	3	4	5	
subitem not at all important	◉	○	○	○	○	essential

Clear selection

Does your paper address subitem 11a-ii?

Copy and paste relevant sections from the manuscript (include quotes in quotation marks "like this" to indicate direct quotes from your manuscript), or elaborate on this item by providing additional information not in the ms, or briefly explain why the item is not applicable/relevant for your study

Both interventions were active arms

11b) If relevant, description of the similarity of interventions

(this item is usually not relevant for ehealth trials as it refers to similarity of a placebo or sham intervention to a active medication/intervention)

Does your paper address CONSORT subitem 11b? *

Copy and paste relevant sections from the manuscript (include quotes in quotation marks "like this" to indicate direct quotes from your manuscript), or elaborate on this item by providing additional information not in the ms, or briefly explain why the item is not applicable/relevant for your study

Not relevant; CBT and IPT have been previously described

12a) Statistical methods used to compare groups for primary and secondary outcomes

NPT: When applicable, details of whether and how the clustering by care providers or centers was addressed

Does your paper address CONSORT subitem 12a? *

Copy and paste relevant sections from the manuscript (include quotes in quotation marks "like this" to indicate direct quotes from your manuscript), or elaborate on this item by providing additional information not in the ms, or briefly explain why the item is not applicable/relevant for your study

"To describe changes in clinical health-related outcomes, change scores from baseline to 12-week follow-up and baseline to one-year follow-up were examined in each arm (CBT and IPT) separately, and this analysis included participants who completed both time points needed for calculation of the change score (i.e., baseline and 12 week; baseline and 1 year). Given the pilot nature of the study, in which we were focused on the estimation of a signal, a one-sample t-test was used to compare to a non-zero benchmark. One-sample t-tests were used for normally distributed outcomes, and Wilcoxon signed rank tests were used when outcomes deviated from normality. Cohen's d was calculated to describe effect sizes for normally distributed outcomes, while r was used for non-normal outcomes. For acceptability ratings, Mann-Whitney tests were used to test for potential group differences across domains of acceptability."

12a-i) Imputation techniques to deal with attrition / missing values

Imputation techniques to deal with attrition / missing values: Not all participants will use the intervention/comparator as intended and attrition is typically high in ehealth trials. Specify how participants who did not use the application or dropped out from the trial were treated in the statistical analysis (a complete case analysis is strongly discouraged, and simple imputation techniques such as LOCF may also be problematic [4]).

	1	2	3	4	5	
subitem not at all important	○	◉	○	○	○	essential

Clear selection

Does your paper address subitem 12a-i? *

Copy and paste relevant sections from the manuscript (include quotes in quotation marks "like this" to indicate direct quotes from your manuscript), or elaborate on this item by providing additional information not in the ms, or briefly explain why the item is not applicable/relevant for your study

"Multiple imputation was not used given that it relies on patterns in the data and therefore may be noisy or unreliable given the small sample size per group [74]."

12b) Methods for additional analyses, such as subgroup analyses and adjusted analyses

Does your paper address CONSORT subitem 12b? *

Copy and paste relevant sections from the manuscript (include quotes in quotation marks "like this" to indicate direct quotes from your manuscript), or elaborate on this item by providing additional information not in the ms, or briefly explain why the item is not applicable/relevant for your study

Not applicable; all analyses were described above.

X26) REB/IRB Approval and Ethical Considerations [recommended as subheading under "Methods"] (not a CONSORT item)

X26-i) Comment on ethics committee approval

	1	2	3	4	5	
subitem not at all important	○	○	○	○	◉	essential

Clear selection

Does your paper address subitem X26-i?

Copy and paste relevant sections from the manuscript (include quotes in quotation marks "like this" to indicate direct quotes from your manuscript), or elaborate on this item by providing additional information not in the ms, or briefly explain why the item is not applicable/relevant for your study

"The use of human subjects in this research was reviewed and approved by the Uniformed Services University Investigative Review Board (IRB #: USUHS.2020-048). Adolescents and parents/guardians were provided with informed consent and assent documents by trained study staff in the first screening visit. Informed consent documents included information on the nature and purpose of the study, study procedures including as at home device wear and group interventions, confidentiality, study benefits, compensation, study risks, alternative therapy programs, and contact information if concerns arose. Adolescents were compensated for participating in various aspects of the study; in total, they could earn up to $917 if they completed all visits, at home data collection, surveys, group sessions, and follow up visits. All data collected were de-identified.
Study staff who administered surveys were trained by a psychologist in safety and risk assessment. Any participants who expressed active self-harm or suicidal ideation were referred to crisis numbers and provided with counseling referrals. Adverse events were monitored by the study principal investigator (blinded for review) and the site principal investigators in conjunction with oversight from an external Safety Officer and study physician (blinded for review). Adverse events were promptly reported to the principal investigators, and any serious adverse events were immediately reported to the USU IRB. The investigators prepared a quarterly report for Data Safety Officer with enrollment, retention, adverse events, and deviations Following recommended guidelines, serious and/or unanticipated adverse events were reported within 5 business days to the IRB and the Data Safety Officer, The safety officer regularly reviewed the data and safety monitoring report and, if needed, recommended corrective action or reporting of out-of-range laboratory data to the participant and their parents/guardians."

x26-ii) Outline informed consent procedures

Outline informed consent procedures e.g., if consent was obtained offline or online (how? Checkbox, etc.?), and what information was provided (see 4a-ii). See [6] for some items to be included in informed consent documents.

	1	2	3	4	5	
subitem not at all important	○	○	○	○	◉	essential

Clear selection

Does your paper address subitem X26-ii?

Copy and paste relevant sections from the manuscript (include quotes in quotation marks "like this" to indicate direct quotes from your manuscript), or elaborate on this item by providing additional information not in the ms, or briefly explain why the item is not applicable/relevant for your study

"Adolescents and parents/guardians were provided with informed consent and assent documents by trained study staff in the first screening visit. Informed consent documents included information on the nature and purpose of the study, study procedures including as at home device wear and group interventions, confidentiality, study benefits, compensation, study risks, alternative therapy programs, and contact information if concerns arose."

X26-iii) Safety and security procedures

Safety and security procedures, incl. privacy considerations, and any steps taken to reduce the likelihood or detection of harm (e.g., education and training, availability of a hotline)

	1	2	3	4	5	
subitem not at all important	○	○	○	◉	○	essential

Clear selection

Does your paper address subitem X26-iii?

Copy and paste relevant sections from the manuscript (include quotes in quotation marks "like this" to indicate direct quotes from your manuscript), or elaborate on this item by providing additional information not in the ms, or briefly explain why the item is not applicable/relevant for your study

"All data collected were de-identified. Study staff who administered surveys were trained by a psychologist in safety and risk assessment. Any participants who expressed active self-harm or suicidal ideation were referred to crisis numbers and provided with counseling referrals. Adverse events were monitored by the study principal investigator (blinded for review) and the site principal investigators in conjunction with oversight from an external Safety Officer and study physician (blinded for review). Adverse events were promptly reported to the principal investigators, and any serious adverse events were immediately reported to the USU IRB. The investigators prepared a quarterly report for Data Safety Officer with enrollment, retention, adverse events, and deviations Following recommended guidelines, serious and/or unanticipated adverse events were reported within 5 business days to the IRB and the Data Safety Officer, The safety officer regularly reviewed the data and safety monitoring report and, if needed, recommended corrective action or reporting of out-of-range laboratory data to the participant and their parents/guardians."

RESULTS

13a) For each group, the numbers of participants who were randomly assigned, received intended treatment, and were analysed for the primary outcome
NPT: The number of care providers or centers performing the intervention in each group and the number of patients treated by each care provider in each center

Does your paper address CONSORT subitem 13a? *

Copy and paste relevant sections from the manuscript (include quotes in quotation marks "like this" to indicate direct quotes from your manuscript), or elaborate on this item by providing additional information not in the ms, or briefly explain why the item is not applicable/relevant for your study

"Recruitment occurred from February 2021 to August 2021, with the total target enrollment of 40 reached in 7 months, corresponding to a rate of 5–6 enrolled participants per month across two sites. Of the 47 adolescents eligible after baseline screening, 79% chose to enroll. CONSORT study flow is depicted in Figure 1 (retention feasibility). Eighty-five percent (17/20) of participants randomized to IPT completed the 12-week follow-up; despite 100% attendance in groups, one participant was unable to complete the 12-week follow-up due to scheduling conflicts. 100% (20/20) of those randomized to CBT completed the 12-week follow-up. At one-year follow-up, 85% (17/20) of IPT participants and 85% (17/20) of CBT participants completed follow-up."

13b) For each group, losses and exclusions after randomisation, together with reasons

Does your paper address CONSORT subitem 13b? (NOTE: Preferably, this is shown in a CONSORT flow diagram) *

Copy and paste relevant sections from the manuscript (include quotes in quotation marks "like this" to indicate direct quotes from your manuscript), or elaborate on this item by providing additional information not in the ms, or briefly explain why the item is not applicable/relevant for your study

"Eighty-five percent (17/20) of participants randomized to IPT completed the 12-week follow-up; despite 100% attendance in groups, one participant was unable to complete the 12-week follow-up due to scheduling conflicts."

13b-i) Attrition diagram

Strongly recommended: An attrition diagram (e.g., proportion of participants still logging in or using the intervention/comparator in each group plotted over time, similar to a survival curve) or other figures or tables demonstrating usage/dose/engagement.

	1	2	3	4	5	
subitem not at all important	○	○	○	○	◉	essential

Clear selection

Does your paper address subitem 13b-i?

Copy and paste relevant sections from the manuscript or cite the figure number if applicable (include quotes in quotation marks "like this" to indicate direct quotes from your manuscript), or elaborate on this item by providing additional information not in the ms, or briefly explain why the item is not applicable/relevant for your study

This is denoted as Figure 1: Consort Flow Diagram

14a) Dates defining the periods of recruitment and follow-up

Does your paper address CONSORT subitem 14a? *

Copy and paste relevant sections from the manuscript (include quotes in quotation marks "like this" to indicate direct quotes from your manuscript), or elaborate on this item by providing additional information not in the ms, or briefly explain why the item is not applicable/relevant for your study

"Recruitment occurred from February 2021 to August 2021" Follow up occurred for up to one year after group completion.

14a-i) Indicate if critical “secular events” fell into the study period

Indicate if critical “secular events” fell into the study period, e.g., significant changes in Internet resources available or “changes in computer hardware or Internet delivery resources”

	1	2	3	4	5	
subitem not at all important	(●)	○	○	○	○	essential

Clear selection

Does your paper address subitem 14a-i?

Copy and paste relevant sections from the manuscript (include quotes in quotation marks "like this" to indicate direct quotes from your manuscript), or elaborate on this item by providing additional information not in the ms, or briefly explain why the item is not applicable/relevant for your study

Not applicable; no changes in internet resources

14b) Why the trial ended or was stopped (early)

Does your paper address CONSORT subitem 14b? *

Copy and paste relevant sections from the manuscript (include quotes in quotation marks "like this" to indicate direct quotes from your manuscript), or elaborate on this item by providing additional information not in the ms, or briefly explain why the item is not applicable/relevant for your study

Trial was completed after recruitment and interventions were completed.

15) A table showing baseline demographic and clinical characteristics for each group

NPT: When applicable, a description of care providers (case volume, qualification, expertise, etc.) and centers (volume) in each group

Does your paper address CONSORT subitem 15? *

Copy and paste relevant sections from the manuscript (include quotes in quotation marks "like this" to indicate direct quotes from your manuscript), or elaborate on this item by providing additional information not in the ms, or briefly explain why the item is not applicable/relevant for your study

This is Table 1 (pages 18-19)

15-i) Report demographics associated with digital divide issues

In ehealth trials it is particularly important to report demographics associated with digital divide issues, such as age, education, gender, social-economic status, computer/Internet/ehealth literacy of the participants, if known.

	1	2	3	4	5	
subitem not at all important	○	○	●	○	○	essential

Clear selection

Does your paper address subitem 15-i? *

Copy and paste relevant sections from the manuscript (include quotes in quotation marks "like this" to indicate direct quotes from your manuscript), or elaborate on this item by providing additional information not in the ms, or briefly explain why the item is not applicable/relevant for your study

Reported parental highest degree of education and subjective social status.

16) For each group, number of participants (denominator) included in each analysis and whether the analysis was by original assigned groups

16-i) Report multiple “denominators” and provide definitions

Report multiple “denominators” and provide definitions: Report N’s (and effect sizes) “across a range of study participation [and use] thresholds” [1], e.g., N exposed, N consented, N used more than x times, N used more than y weeks, N participants “used” the intervention/comparator at specific pre-defined time points of interest (in absolute and relative numbers per group). Always clearly define “use” of the intervention.

	1	2	3	4	5	
subitem not at all important	○	○	○	◉	○	essential

Clear selection

Does your paper address subitem 16-i? *

Copy and paste relevant sections from the manuscript (include quotes in quotation marks "like this" to indicate direct quotes from your manuscript), or elaborate on this item by providing additional information not in the ms, or briefly explain why the item is not applicable/relevant for your study

"Figure 2 shows average acceptability ratings by arm. Nonparametric tests of group differences revealed higher ratings of the helpfulness of at home activities in the IPT group (mean rank 19.22 in IPT versus 15.97 in CBT, p = .02). There was also a trend where IPT participants were more likely to report that they would participate in the program if they could do it over, (mean rank 20.59 versus 14.75 p = .07). All Mann-Whitney comparisons should be interpreted with caution, however, given the small sample size.
Descriptively, In CBT, 61.1% (11/18) would participate again, 72.2% (13/18) found the group “mostly” or “extremely” enjoyable, and 77.8% (14/18) thought it would be “mostly” or “extremely” helpful for peers. The majority felt “very” or “extremely” comfortable sharing (72.2%, 13/18), perceived group leaders (94.4%, 17/18) and members (83.3%, 15/18) as supportive, and found activities “very” or “extremely” helpful (72.2%, 13/18). Only 38.9% (7/18) found home practice exercises “very” or “extremely” helpful.
In IPT, 87.5% (14/16) of adolescents reported they would “mostly” or “definitely” complete the program again (3 or 4 on a 0–4 scale), 87.5% (14/16) found the group “very” to “definitely” enjoyable, and 87.5% (14/16) thought the group would be “mostly” or “extremely” helpful for peers. Most adolescents in IPT felt “very” or “extremely” comfortable sharing and asking questions (75.0%, 12/16), viewed facilitators (93.8%, 15/16) and group members (93.8%, 15/16) as supportive, found group activities “very” or “extremely” helpful (71.3%, 13/16), and saw home practice exercises as “very” or “extremely” useful (75.0%, 12/16).
In the intent-to-treat sample, 90% (18/20) in IPT and 95% (19/20) in CBT received ≥80% of the intervention dose (≥10 of 12 group sessions). Median attendance was 12 sessions in CBT and 11 in IPT. One IPT participant withdrew prior to starting due to loss of interest, and one was withdrawn due to an expected, serious adverse event unrelated to the protocol: worsening mental health symptoms that necessitated hospitalization and precluded ongoing research participation. Excluding withdrawn participants, mean attendance was 11.55 (SD 1.36) sessions in CBT and 11.00 (SD 1.46) sessions in IPT and.
Results: Secondary Aims
Key clinical outcomes at each interval by arm are detailed in Table 3. Change scores in anxiety, disinhibited eating, BMI indices and adiposity, as well as cardiometabolic health indices and within-arm effect sizes are shown in Table 4. From baseline to post-intervention/12-week follow-up, adolescents in both CBT and IPT experienced statistically significant improvements in emotional eating. For adolescents enrolled in CBT, no changes were observed in social anxiety, loss-of-control eating, BMI, BMI percentile, fat mass, blood pressure, triglycerides, cholesterol, or glucose; however, HbA1c significantly increased from baseline to 12-week follow-up. No significant changes were observed in social anxiety, loss-of-control eating, BMI, fat mass, blood pressure, triglycerides, cholesterol, HbA1c, or glucose from baseline to 12-week follow-up for adolescents enrolled in IPT. BMI percentile significantly decreased in the IPT group."

16-ii) Primary analysis should be intent-to-treat

Primary analysis should be intent-to-treat, secondary analyses could include comparing only "users", with the appropriate caveats that this is no longer a randomized sample (see 18-i).

	1	2	3	4	5	
subitem not at all important	○	○	○	●	○	essential

Clear selection

Does your paper address subitem 16-ii?

Copy and paste relevant sections from the manuscript (include quotes in quotation marks "like this" to indicate direct quotes from your manuscript), or elaborate on this item by providing additional information not in the ms, or briefly explain why the item is not applicable/relevant for your study

"In the intent-to-treat sample, 90% (18/20) in IPT and 95% (19/20) in CBT received ≥80% of the intervention dose (≥10 of 12 group sessions). Median attendance was 12 sessions in CBT and 11 in IPT. One IPT participant withdrew prior to starting due to loss of interest, and one was withdrawn due to an expected, serious adverse event unrelated to the protocol: worsening mental health symptoms that necessitated hospitalization and precluded ongoing research participation. Excluding withdrawn participants, mean attendance was 11.55 (SD 1.36) sessions in CBT and 11.00 (SD 1.46) sessions in IPT."

17a) For each primary and secondary outcome, results for each group, and the estimated effect size and its precision (such as 95% confidence interval)

Does your paper address CONSORT subitem 17a? *

Copy and paste relevant sections from the manuscript (include quotes in quotation marks "like this" to indicate direct quotes from your manuscript), or elaborate on this item by providing additional information not in the ms, or briefly explain why the item is not applicable/relevant for your study

95% CIs, effect size ranged from 0.00 (Loss of Control Eating) to 1.05 (Emotional Eating), see Table 4

17a-i) Presentation of process outcomes such as metrics of use and intensity of use

In addition to primary/secondary (clinical) outcomes, the presentation of process outcomes such as metrics of use and intensity of use (dose, exposure) and their operational definitions is critical. This does not only refer to metrics of attrition (13-b) (often a binary variable), but also to more continuous exposure metrics such as "average session length". These must be accompanied by a technical description how a metric like a "session" is defined (e.g., timeout after idle time) [1] (report under item 6a).

	1	2	3	4	5	
subitem not at all important	○	○	○	○	◉	essential

Clear selection

Does your paper address subitem 17a-i?

Copy and paste relevant sections from the manuscript (include quotes in quotation marks "like this" to indicate direct quotes from your manuscript), or elaborate on this item by providing additional information not in the ms, or briefly explain why the item is not applicable/relevant for your study

Sessions were 90 minutes and occurred weekly. "Median attendance was 12 sessions in CBT and 11 in IPT. One IPT participant withdrew prior to starting due to loss of interest, and one was withdrawn due to an expected, serious adverse event unrelated to the protocol: worsening mental health symptoms that necessitated hospitalization and precluded ongoing research participation. Excluding withdrawn participants, mean attendance was 11.55 (SD 1.36) sessions in CBT and 11.00 (SD 1.46) sessions in IPT."

17b) For binary outcomes, presentation of both absolute and relative effect sizes is recommended

Does your paper address CONSORT subitem 17b? *

Copy and paste relevant sections from the manuscript (include quotes in quotation marks "like this" to indicate direct quotes from your manuscript), or elaborate on this item by providing additional information not in the ms, or briefly explain why the item is not applicable/relevant for your study

Not applicable; no binary outcomes

18) Results of any other analyses performed, including subgroup analyses and adjusted analyses, distinguishing pre-specified from exploratory

Does your paper address CONSORT subitem 18? *

Copy and paste relevant sections from the manuscript (include quotes in quotation marks "like this" to indicate direct quotes from your manuscript), or elaborate on this item by providing additional information not in the ms, or briefly explain why the item is not applicable/relevant for your study

Not applicable; primary and secondary analyses already discussed

18-i) Subgroup analysis of comparing only users

A subgroup analysis of comparing only users is not uncommon in ehealth trials, but if done, it must be stressed that this is a self-selected sample and no longer an unbiased sample from a randomized trial (see 16-iii).

	1	2	3	4	5	
subitem not at all important	(●)	○	○	○	○	essential

Clear selection

Does your paper address subitem 18-i?

Copy and paste relevant sections from the manuscript (include quotes in quotation marks "like this" to indicate direct quotes from your manuscript), or elaborate on this item by providing additional information not in the ms, or briefly explain why the item is not applicable/relevant for your study

Not applicable; no subgroup analyses

19) All important harms or unintended effects in each group

(for specific guidance see CONSORT for harms)

Does your paper address CONSORT subitem 19? *

Copy and paste relevant sections from the manuscript (include quotes in quotation marks "like this" to indicate direct quotes from your manuscript), or elaborate on this item by providing additional information not in the ms, or briefly explain why the item is not applicable/relevant for your study

Not applicable; no harms or unintended effects

19-i) Include privacy breaches, technical problems

Include privacy breaches, technical problems. This does not only include physical "harm" to participants, but also incidents such as perceived or real privacy breaches [1], technical problems, and other unexpected/unintended incidents. "Unintended effects" also includes unintended positive effects [2].

	1	2	3	4	5	
subitem not at all important	(●)	( )	( )	( )	( )	essential

Clear selection

Does your paper address subitem 19-i?

Copy and paste relevant sections from the manuscript (include quotes in quotation marks "like this" to indicate direct quotes from your manuscript), or elaborate on this item by providing additional information not in the ms, or briefly explain why the item is not applicable/relevant for your study

Not applicable; no privacy breaches or technical problems

19-ii) Include qualitative feedback from participants or observations from staff/researchers

Include qualitative feedback from participants or observations from staff/researchers, if available, on strengths and shortcomings of the application, especially if they point to unintended/unexpected effects or uses. This includes (if available) reasons for why people did or did not use the application as intended by the developers.

	1	2	3	4	5	
subitem not at all important	(selected)	○	○	○	○	essential

Clear selection

Does your paper address subitem 19-ii?

Copy and paste relevant sections from the manuscript (include quotes in quotation marks "like this" to indicate direct quotes from your manuscript), or elaborate on this item by providing additional information not in the ms, or briefly explain why the item is not applicable/relevant for your study

Not applicable; did not include qualitative feedback in this RCT.

DISCUSSION

22) Interpretation consistent with results, balancing benefits and harms, and considering other relevant evidence

NPT: In addition, take into account the choice of the comparator, lack of or partial blinding, and unequal expertise of care providers or centers in each group

22-i) Restate study questions and summarize the answers suggested by the data, starting with primary outcomes and process outcomes (use)

Restate study questions and summarize the answers suggested by the data, starting with primary outcomes and process outcomes (use).

	1	2	3	4	5	
subitem not at all important	○	○	○	○	◉	essential

Clear selection

Does your paper address subitem 22-i? *

Copy and paste relevant sections from the manuscript (include quotes in quotation marks "like this" to indicate direct quotes from your manuscript), or elaborate on this item by providing additional information not in the ms, or briefly explain why the item is not applicable/relevant for your study

"This study was the first to directly pilot the feasibility, acceptability, and exploratory outcomes on anxiety and cardiometabolic health markers of web-based, group CBT and IPT for adolescent girls with above average weight and elevated anxiety symptoms. The target sample size of 40 adolescents was achieved across two sites in 7 months, with an enrollment rate of 5–6 participants per month. Of eligible adolescents, 79% elected to enroll. Retention exceeded 80% at 12-week follow-up and 70% at one-year follow-up. Specifically, at 12-week follow up, 95% of participants from USU, and 90% of participants at CSU completed the post-treatment visit (Chi sq p =.55). At one-year follow up, 85% of participants at both sites were retained (Chi sq p = 1.0), Protocol adherence by research staff was high (>96%) across time points. Data missingness ranged from 0% to 30%, with no missing height/weight data. Questionnaire missingness ranged from 0% to 15%, and interview and body measurements missingness never exceeded 5.9%. Cardiometabolic measures requiring phlebotomy had the greatest missingness (15%–25% at baseline, 11.8%–30% at follow-up). Intervention fidelity ratings indicated high CBT adherence (>90%); IPT adherence was just below the 80% threshold. Overall acceptability was high, with >90% receiving at least 80% of intervention dosage in both arms. However, home practice assignments were rated as the least helpful aspect of both IPT and CBT web-based group interventions."

22-ii) Highlight unanswered new questions, suggest future research

Highlight unanswered new questions, suggest future research.

	1	2	3	4	5	
subitem not at all important	○	○	○	○	●	essential

Clear selection

Does your paper address subitem 22-ii?

Copy and paste relevant sections from the manuscript (include quotes in quotation marks "like this" to indicate direct quotes from your manuscript), or elaborate on this item by providing additional information not in the ms, or briefly explain why the item is not applicable/relevant for your study

"Future fully powered trials should be designed to test comparative efficacy between IPT and CBT, using a prespecified primary endpoint and adequate sample size. In the present pilot, within-arm effect sizes for key outcomes were generally in the moderate-to-large range. For planning purposes, anchoring to a moderate effect (d = 0.50; α = .05, 80% power), a future trial would require approximately 34 participants per arm with complete data, corresponding to approximately 43-49 participants per arm after accounting for ~20-30% attrition and biospecimen missingness. Larger samples would be required to adequately detect between-group differences in treatment response. Furthermore, future research should engage adolescent interest holders to identify barriers and facilitators to homework adherence and explore digital or technological supports as enhancement to web-based group interventions
Therefore, areas of refinement include phlebotomy procedures, specifically, planning for larger sample sizes to accommodate biospecimen missingness and prospectively documenting reasons for missed or unsuccessful blood draws, IPT training/supervision related to adherence and competence, sufficient retention to confirm the impact of CBT and IPT on cardiometabolic health for adolescent girls, and including non-active control group. Specifically, future trials should anticipate missing data from phlebotomy and consider specialized personnel or noninvasive alternatives (e.g., ultrasound) to reduce missingness; use of finger pricks or non-fasting measures would also be expected to improve data completeness (Hjelmgren et al., 2021). Fully powered efficacy trials should also aim to assess the mediational pathways of the mechanisms of both CBT and IPT, evaluate generalized anxiety at all follow-up intervals to better characterize trajectories of anxiety, plan for greater missingness in power estimations, and account for pubertal changes during the follow-up period. Additionally, future trials should test the impact of interventions for girls with more frequent loss of control eating and/or binge eating disorder.
Given the high prevalence of anxiety among adolescent girls with excess weight and evidence that mental health interventions can improve behavioral and cardiometabolic outcomes, targeting anxiety represents a promising prevention strategy. Web-based group-based interventions may offer a scalable and accessible approach to addressing these intersecting risks and will benefit from continued interdisciplinary collaboration across psychology, pediatrics, and cardiometabolic health. Building on the feasibility and acceptability demonstrated in this pilot, a fully powered randomized trial is warranted to evaluate the efficacy and mechanisms of virtual group IPT and CBT in this population."

20) Trial limitations, addressing sources of potential bias, imprecision, and, if relevant, multiplicity of analyses

20-i) Typical limitations in ehealth trials

Typical limitations in ehealth trials: Participants in ehealth trials are rarely blinded. Ehealth trials often look at a multiplicity of outcomes, increasing risk for a Type I error. Discuss biases due to non-use of the intervention/usability issues, biases through informed consent procedures, unexpected events.

	1	2	3	4	5	
subitem not at all important	○	○	○	○	●	essential

Clear selection

Does your paper address subitem 20-i? *

Copy and paste relevant sections from the manuscript (include quotes in quotation marks "like this" to indicate direct quotes from your manuscript), or elaborate on this item by providing additional information not in the ms, or briefly explain why the item is not applicable/relevant for your study

"Limitations include (1) limited power, (2) high proportion of missing data for outcomes collected via phlebotomy, (3) omission of the general anxiety symptom assessment at 12-week follow-up, and (4) lack of controlling for pubertal stage of development. Notably, the study was not powered to assess comparative effects; within-arm changes should be interpreted cautiously given the small sample size. Likewise, as this study was a pilot of two active interventions without an inactive control arm, any within-arm improvements cannot be attributed to the specific modalities tested; it is possible that changes were influenced by natural maturation, non-specific therapeutic attention effects, and/or regression to the mean.
Moreover, (5) Missing data were not uniform across outcomes and were concentrated in phlebotomy-derived cardiometabolic measures. Whereas questionnaire, interview, and anthropometric outcomes were largely complete, blood-based measures had 15%-30%

missingness across arms and timepoints, exceeding the study's a priori feasibility benchmark (6). Accordingly, exploratory findings for HbA1c, glucose, and lipid outcomes should be interpreted cautiously, as missingness likely reduced precision, effective sample size, and power to detect small-to-moderate effects. Because this pilot was not designed or powered to formally evaluate missing-data mechanisms, the extent of potential bias remains uncertain. Exploratory comparisons of participants with versus without follow-up phlebotomy data on baseline characteristics showed no meaningful baseline differences in participants with and without follow-up phlebotomy data, suggesting limited evidence of systematic missingness based on observed variables.
However, the group of participants with missing baseline phlebotomy data had higher baseline BMI (97.7±.64 versus 94.12±4.9, $p < .01$) and a greater proportion of Hispanic/Latina participants (40% versus 6%, Fisher's Exact Test = .03) versus the group with baseline phlebotomy data. Therefore, we cannot rule out the possibility of systematic missingness at baseline based on higher BMI and Hispanic Ethnicity. In future studies will ensure baseline measures of phlebotomy are collected across race/ethnicity groups and BMI strata, particularly in youth with higher BMI and Latina girls, and addressing potential barriers in youth with more severe obesity and Latina girls, is needed.
While social anxiety symptoms were assessed at baseline and all follow-up intervals, general anxiety symptoms only were assessed at baseline and 1-year as part of an effort to limit participant questionnaire fatigue and given the study's focus on feasibility/acceptability as primary outcomes (7). Also, with respect to measurement, (8) Tanner pubertal stage was not evaluated. Additional limitations include that (9) fidelity assessment focused on adherence, not competence and that (10) homework completion was not objectively measured. Participants could not be blinded (11); the multiplicity of outcomes increases risk for Type 1 Error. The absence of qualitative data limits participant perspective on intervention experience (12). Additionally, the sample was demographically homogenous (13), thereby limiting external validity and generalizability of the feasibility and acceptability findings to lower-income or minority populations. Reductions in loss-of-control eating episodes must be considered cautiously (14), as not all adolescents had loss-of-control at baseline and frequencies of loss-of-control episodes were low."

21) Generalisability (external validity, applicability) of the trial findings

NPT: External validity of the trial findings according to the intervention, comparators, patients, and care providers or centers involved in the trial

21-i) Generalizability to other populations

Generalizability to other populations: In particular, discuss generalizability to a general Internet population, outside of a RCT setting, and general patient population, including applicability of the study results for other organizations

	1	2	3	4	5	
subitem not at all important	(●)	( )	( )	( )	( )	essential

Clear selection

Does your paper address subitem 21-i?

Copy and paste relevant sections from the manuscript (include quotes in quotation marks "like this" to indicate direct quotes from your manuscript), or elaborate on this item by providing additional information not in the ms, or briefly explain why the item is not applicable/relevant for your study

Not applicable; pilot and feasibility trial

21-ii) Discuss if there were elements in the RCT that would be different in a routine application setting

Discuss if there were elements in the RCT that would be different in a routine application setting (e.g., prompts/reminders, more human involvement, training sessions or other co-interventions) and what impact the omission of these elements could have on use, adoption, or outcomes if the intervention is applied outside of a RCT setting.

	1	2	3	4	5	
subitem not at all important	(●)	○	○	○	○	essential

Clear selection

Does your paper address subitem 21-ii?

Copy and paste relevant sections from the manuscript (include quotes in quotation marks "like this" to indicate direct quotes from your manuscript), or elaborate on this item by providing additional information not in the ms, or briefly explain why the item is not applicable/relevant for your study

Not applicable; pilot and feasibility trial, not yet in efficacy and effectiveness testing stage

OTHER INFORMATION

23) Registration number and name of trial registry

Does your paper address CONSORT subitem 23? *

Copy and paste relevant sections from the manuscript (include quotes in quotation marks "like this" to indicate direct quotes from your manuscript), or elaborate on this item by providing additional information not in the ms, or briefly explain why the item is not applicable/relevant for your study

https://clinicaltrials.gov/study/NCT05038033

24) Where the full trial protocol can be accessed, if available

Does your paper address CONSORT subitem 24? *

Cite a Multimedia Appendix, other reference, or copy and paste relevant sections from the manuscript (include quotes in quotation marks "like this" to indicate direct quotes from your manuscript), or elaborate on this item by providing additional information not in the ms, or briefly explain why the item is not applicable/relevant for your study

https://clinicaltrials.gov/study/NCT05038033

25) Sources of funding and other support (such as supply of drugs), role of funders

Does your paper address CONSORT subitem 25? *

Copy and paste relevant sections from the manuscript (include quotes in quotation marks "like this" to indicate direct quotes from your manuscript), or elaborate on this item by providing additional information not in the ms, or briefly explain why the item is not applicable/relevant for your study

Funding for this study was provided by Defense Health Agency grants HU000119200029 and HU00012420018 (Haigney). The study was funded for five years for a total of $560,000.

X27) Conflicts of Interest (not a CONSORT item)

X27-i) State the relation of the study team towards the system being evaluated

In addition to the usual declaration of interests (financial or otherwise), also state the relation of the study team towards the system being evaluated, i.e., state if the authors/evaluators are distinct from or identical with the developers/sponsors of the intervention.

	1	2	3	4	5	
subitem not at all important	○	○	○	●	○	essential

Clear selection

Does your paper address subitem X27-i?

Copy and paste relevant sections from the manuscript (include quotes in quotation marks "like this" to indicate direct quotes from your manuscript), or elaborate on this item by providing additional information not in the ms, or briefly explain why the item is not applicable/relevant for your study

The authors have no conflicts of interest to disclose. Authors/evaluators are distinct from the developers of CBT and IPT.

About the CONSORT EHEALTH checklist

As a result of using this checklist, did you make changes in your manuscript? *

- ○ yes, major changes
- ◉ yes, minor changes
- ○ no

What were the most important changes you made as a result of using this checklist?

Your answer

How much time did you spend on going through the checklist INCLUDING making changes in your manuscript *

A minimum of 90 minutes up to 2 hours

As a result of using this checklist, do you think your manuscript has improved? *

- (●) yes
- ( ) no
- ( ) Other:

Would you like to become involved in the CONSORT EHEALTH group?

This would involve for example becoming involved in participating in a workshop and writing an "Explanation and Elaboration" document

- (●) yes
- ( ) no
- ( ) Other:

Clear selection

Any other comments or questions on CONSORT EHEALTH

Your answer

STOP - Save this form as PDF before you click submit

To generate a record that you filled in this form, we recommend to generate a PDF of this page (on a Mac, simply select "print" and then select "print as PDF") before you submit it.

When you submit your (revised) paper to JMIR, please upload the PDF as supplementary file.

Don't worry if some text in the textboxes is cut off, as we still have the complete information in our database. Thank you!

Final step: Click submit !

Click submit so we have your answers in our database!

Submit

Clear form

Never submit passwords through Google Forms.

This content is neither created nor endorsed by Google. - Terms of Service - Privacy Policy

Does this form look suspicious? Report

Google Forms
